# MiR-1908/EXO1 and MiR-203a/FOS, regulated by scd1, are associated with fracture risk and bone health in postmenopausal diabetic women

**DOI:** 10.18632/aging.103227

**Published:** 2020-05-26

**Authors:** Yi-sheng Chen, Xue-ran Kang, Zi-hui Zhou, Jiang Yang, Qi Xin, Chen-ting Ying, Yun-peng Zhang, Jie Tao

**Affiliations:** 1 Department of Orthopedics, Shanghai General Hospital, Shanghai Jiao Tong University School of Medicine, Shanghai Jiao Tong University, Shanghai 200080, China; 2 Department of Otolaryngology-Head and Neck Surgery, Shanghai Ninth People’s Hospital, Shanghai Jiao Tong University School of Medicine, Ear Institute, Shanghai Jiao Tong University School of Medicine, Shanghai 200080, China; 3 Department of Neurosurgery, Shanghai General Hospital, Shanghai Jiao Tong University School of Medicine, Shanghai Jiao Tong University, Shanghai 200080, China

**Keywords:** fracture risk, bone mesenchymal stem cell (BMSC), stearoyl-coenzyme A desaturase (SCD1), diabetes, nomogram

## Abstract

Background: Stearoyl–coenzyme A desaturase-1 (SCD1) can inhibit the development of diabetic bone disease by promoting osteogenesis. In this study, we examined whether this regulation by SCD1 is achieved by regulating the expression of related miRNAs.

Methods: SCD1 expression levels were observed in human bone-marrow mesenchymal stem cells (BM-MSCs) of patients with type 2 diabetes mellitus (T2DM), and the effect of SCD1 on osteogenesis was observed in human adipose-derived MSCs transfected with the SCD1 lentiviral system. We designed a bioinformatics prediction model to select important differentially expressed miRNAs, and established protein–protein interaction and miRNA–mRNA networks. miRNAs and mRNAs were extracted and their differential expression was detected. The SCD1–miRNA–mRNA network was validated.

Findings: SCD1 expression in bone marrow was downregulated in patients with T2DM and low-energy fracture, and SCD1 expression promotes BM-MSC osteogenic differentiation. The predictors in the nomogram were seven microRNAs, including hsa-miR-1908 and hsa-miR-203a. SCD1 inhibited the expression of CDKN1A and FOS, but promoted the expression of EXO1 and PLS1. miR-1908 was a regulator of EXO1 expression, and miR-203a was a regulator of FOS expression.

Interpretation: The regulation of BM-MSCs by SCD1 is a necessary condition for osteogenesis through the miR-203a/FOS and miR-1908/EXO1 regulatory pathways.

## INTRODUCTION

Diabetes mellitus (DM) affected 158.8 million people aged 20–79 years in the Western Pacific region in 2017, translating to a 9.5% prevalence rate [1]. As many cases of fracture are reported in patients with DM [2–7]. Mechanistic studies are required to determine the cause of the decline in bone health in this disease context.

Bone remodeling depends on bone formation by osteoblasts are formed by bone marrow–derived mesenchymal stem cells (BM-MSCs) [8]. BM-MSCs are key modulators of anti-inflammatory, anti-apoptotic, and angiogenic processes. [9] In a rat model of type 2 diabetes mellitus (T2DM), insufficient differentiation of MSCs into osteoblasts led to inflammation that impaired fracture healing, and metformin promoted this differentiation [10, 11]. However, the way in which hyperglycemia disturbs the bone MSC microenvironment and the molecular mechanism underlying inflammatory response upregulation remain largely unknown.

Our previous research revealed that stearoyl–coenzyme A desaturase-1 (SCD1), an enzyme responsible for the addition of unsaturation bonds to the fatty acid precursors of stearate and palmitate [12–17], enhanced osteogenesis by promoting osteogenic differentiation of MSCs and oxidative stress [18, 19]. Compared with healthy controls, postmenopausal patients with osteoporosis have increased levels of let-7c [19]. *In vitro* experiments showed that the overexpression of let-7c inhibited osteogenic differentiation, and the inhibition of let-7c function promoted this process. A luciferase reporter assay verified that let-7c is a target molecule of SCD1, and the silencing of SCD1 significantly reduced the effects of let-7c inhibitors on osteoblast markers [19]. These data indicate that SCD1 significantly promotes osteogenic differentiation. However, whether patients with T2DM benefit from SCD1 is not clear, as high SCD1 activity has been related to fatty liver and insulin resistance in humans [20]. On the other hand, the incidence of metabolic diseases is reportedly lower in patients with high unsaturated/saturated fat ratios and inflammatory responses [21]. Thus, the question arises of whether high SCD1 expression represents protective factors in the hyperglycemic microenvironment that prevent further development of metabolic disorders.

Messenger RNAs (mRNAs) are regulated by miRNAs, which degrade or inhibit their translation into proteins by interacting with their 3’ untranslated regions [22, 23]. Thus, miRNAs are key factors that fine tune several processes, including oxidative stress, differentiation, remodeling, and apoptosis [24–26]. Diseases such as osteoarthritis [27, 28], T2DM [29], coronary heart disease [30], and cancer [31, 32] are influenced by changes in serum miRNA levels. Recently, numerous dysregulated miRNAs were identified and shown to have major effects on bone metabolism in fracture and diabetes [33, 34]. We showed that let-7c is involved with the translation of MSCs via SCD1 targeting and the reduction of osteogenic transcription factor activation; we also found that SCD1 induced significantly differential expression of several fracture-related miRNAs [19], suggesting the involvement of miRNA/SCD1 networks in bone health. Similarly, a recent study showed that oleic acid (OA), a product of SCD1 catalysis, induces miR-203a expression [35]. Therefore, we speculated that SCD1, as a factor involved in diabetes development, exerts control over bone MSCs required for the proliferation and development of osteocytes in the hyperglycemic bone microenvironment through SCD1/miRNA/mRNA regulatory pathways. The objective of this study was to examine this speculation.

### Evidence before this study

Diabetic fracture is characterized by bone quality deterioration in the hyperglycemic microenvironment. Previous research revealed that stearoyl–coenzyme A desaturase (SCD1), which influences the development of diabetes and enhances osteogenesis, may regulate the expression of micro-RNA (miRNA). However, miRNAs and mRNAs expression pattern after overexpression of SCD1 and the underlying mechanism associated with fracture risk in diabetic patients remains unclear.

### Added value of this study

According to our nomogram prediction model, hsa-miR-550a-5p, hsa-miR-382-3p, hsa-miR-369-3p, hsa-miR-376c-3p, hsa-miR-1908, hsa-miR-203a, and hsa-miR-942 were identified as the predictors of fracture in diabetic patients. This nomogram is suitable for the prediction of fracture risk in diabetic patients. The miRNAs–mRNAs network indicated that the majority of hub genes associated with diabetes were influenced by those predictors. Furthermore, experiments and microarray analyses demonstrated that SCD1 could be beneficial in the treatment of patients with diabetes and high fracture risk and characterized a fracture risk regulatory network involving dysregulated miRNAs and hub genes after SCD1 overexpression in MSCs.

### Implications of all the available evidence

This study showed that SCD1/miR-203a/FOS and SCD1/miR-1908/EXO1 are necessary for bone health. Moreover, the fracture risk nomogram and the miRNAs-mRNAs network identified in this study also provide a basis for further exploration of the therapeutic targets and biomarkers underlying fracture in the context of type 2 diabetes.

Diabetic fracture is characterized by bone quality deterioration in the hyperglycemic microenvironment. Previous research revealed that stearoyl–coenzyme A desaturase (SCD1), which influences the development of diabetes and enhances osteogenesis, may regulate the expression of micro-RNA (miRNA). However, miRNAs and mRNAs expression pattern after overexpression of SCD1 and the underlying mechanism associated with fracture risk in diabetic patients remains unclear.

According to our nomogram prediction model, hsa-miR-550a-5p, hsa-miR-382-3p, hsa-miR-369-3p, hsa-miR-376c-3p, hsa-miR-1908, hsa-miR-203a, and hsa-miR-942 were identified as the predictors of fracture in diabetic patients. This nomogram is suitable for the prediction of fracture risk in diabetic patients. The miRNAs–mRNAs network indicated that the majority of hub genes associated with diabetes were influenced by those predictors. Furthermore, experiments and microarray analyses demonstrated that SCD1 could be beneficial in the treatment of patients with diabetes and high fracture risk and characterized a fracture risk regulatory network involving dysregulated miRNAs and hub genes after SCD1 overexpression in MSCs.

This study showed that SCD1/miR-203a/FOS and SCD1/miR-1908/EXO1 are necessary for bone health. Moreover, the fracture risk nomogram and the miRNAs-mRNAs network identified in this study also provide a basis for further exploration of the therapeutic targets and biomarkers underlying fracture in the context of type 2 diabetes.

## RESULTS

### SCD1 is downregulated in postmenopausal diabetic women with high fracture risk

**Figure 1:**
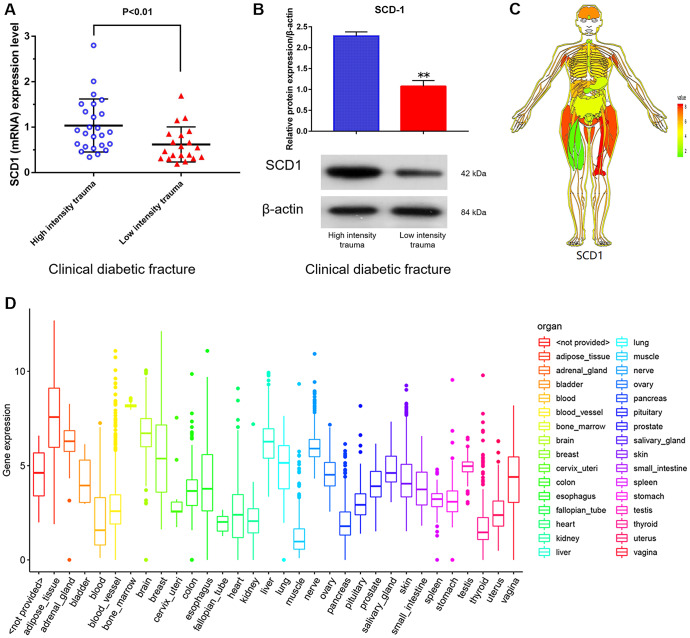
**SCD1 expression in humans.** (**A**) (mRNA) (**B**) (protein) showed the difference in SCD1 expression between patients with diabetic high-intensity fractures and patients with low-intensity fractures. (**C**, **D**) showed the human tissue enriched protein expression maps and the boxplot of SCD1 expression with high levels of SCD1 expression (GTEX cohort, n = 68).

SCD1 expression levels in purified human BM-MSCs from diabetic patients with high-energy fracture (controls) and diabetic patients with low-energy fracture were examined by quantitative real-time PCR. Patients with high-energy fracture (sustained mainly in traffic accidents) were equivalent to the normal control group (diabetic patients with good bone quality and no fracture), and those with low-energy fracture (such as that sustained in falls) were equivalent to the diabetic bone disease group. This grouping was based on ethical considerations, as the collection of bone marrow and bone tissue samples from healthy individuals would not be ethical. The results showed that average SCD1 expression level was significantly lower in diabetic patients with low-energy fracture than in controls (Figure 1A, 1B). To understand the expression of these genes in the human body more intuitively, we constructed human tissue–enriched protein expression maps using the data from the GTEX database (http://www.bio-info-trainee.com/3705.html), which contains bone marrow data from autopsy specimens obtained from subjects who were healthy before death (e.g., traffic accident victims). The results showed that SCD1 was highly enriched in bone (Figure 1C, 1D). Therefore, our findings emphasize the expression of SCD1 may be associated with low-energy fracture risk in diabetics.

### SCD1 overexpression promotes osteogenic differentiation and induces expression changes in BM-MSCs

**Figure 2:**
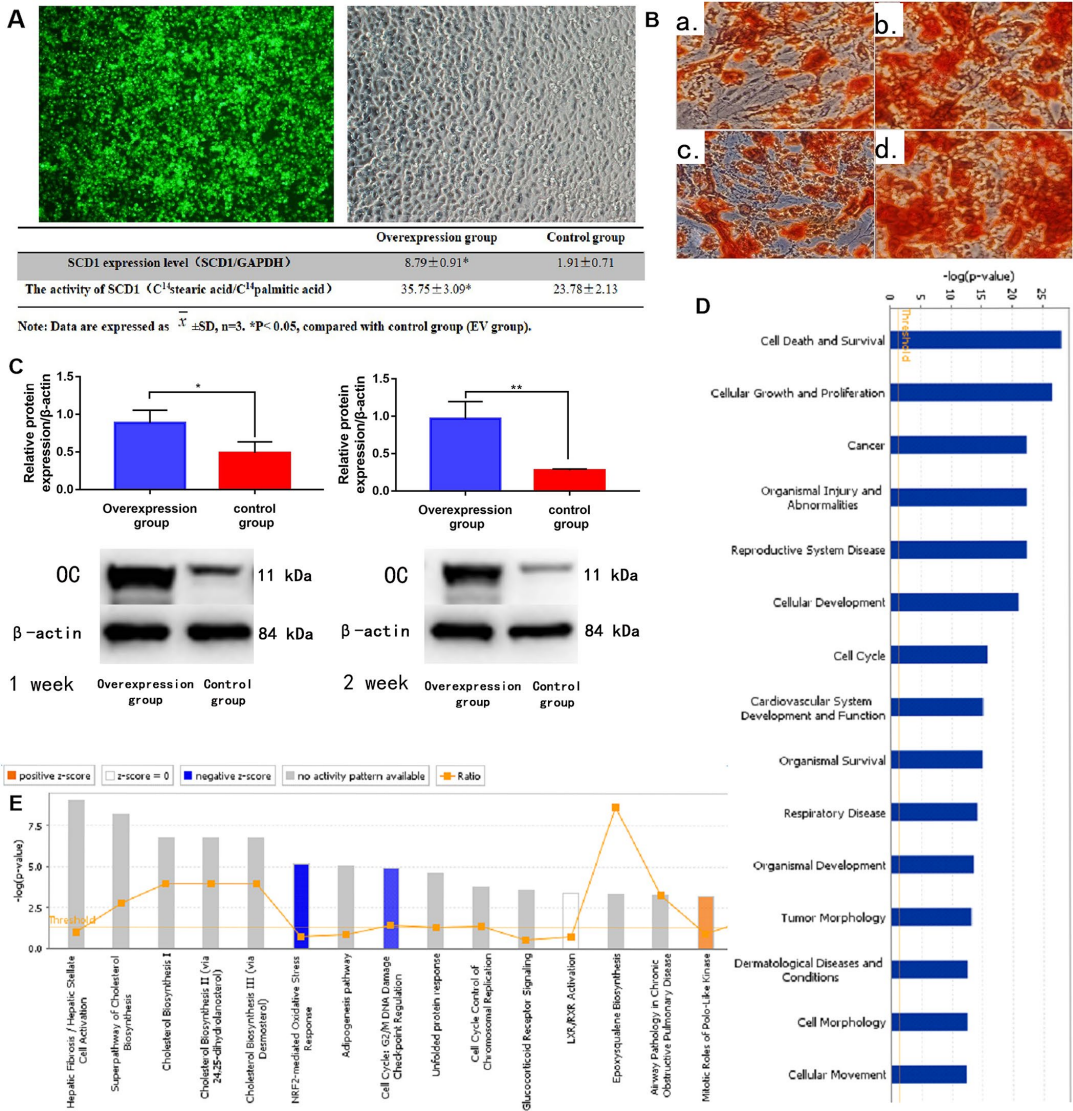
**SCD1 overexpression promotes osteogenic differentiation and induces expression changes in BM-MSCs.** (**A**) Fluorescence staining showed the transfection of BM-MSCs with SCD1 lentivirus. Comparison results of SCD1 expression and activity between the SCD1 overexpression and control groups were showed in the table beneath. Data are expressed as means ± standard deviations (*n* = 6). *P< 0.05 vs. control (EV) group. (**B**) Cetyl staining (a, b: 1-week SCD1 post-transfection; c, d: 2-weeks SCD1 post-transfection; a, c: control; b, d: overexpression). (**C**) Western blot results. (**D**) Signal pathway histogram showing the enrichment of differentially expressed genes in the classical signaling pathway. All signal paths were sorted using –log(*P*); orange, *Z* score>0; blue, *Z* score < 0. *Z* scores > 2 indicate significant pathway activation and *Z* scores < –2 indicate significant pathway inhibition. The ratio of the number of differentially expressed genes to the number of all genes in the signaling pathway is given. The NRF2-mediated oxidative stress response was significantly inhibited, (*Z* score = –21.121). (**E**) Disease and function histograms showing the enrichment of differentially expressed genes. All diseases and functions were sorted using –log(*P*).

BM-MSCs were successfully transfected (at a rate of 88.3%) using the SCD1 lentiviral system (Table 1), as indicated by fluorescence staining (Figure 2A) and signal histogram (Supplementary Figure 1). The RT-PCR analysis revealed higher SCD1 mRNA levels in the SCD1-overexpressing group than in the EV group. SCD1 activity was also significantly greater in transfected BM-MSCs than in the EV group (*P* < 0.05; Figure 2A). These results demonstrate successful construction of an SCD1 overexpression system in BM-MSCs. ALP and cetyl stain activity were significantly greater in SCD1-overexpressing cells than in the EV and control groups at 1 and 2 weeks, suggesting that SCD1 overexpression in BM-MSCs promotes osteogenesis (Figure 2B). Western blot analysis revealed positive correlation between SCD1 and osteocalcin expression levels (Figure 2C), similar to our previous findings. Thus, osteogenesis was more active in SCD1 overexpressing BM-MSCs than in controls.

**Table 1:** The quality control information of RNA sample after overexpression SCD-1.

**Number**	**Sample**	**Thermo NanoDrop 2000**		**2100 results**		**Results**
		**Concentration (ng/μL)**	**A260/A280**	**RIN**	**28S/18S**	
G2017-1	NC	583.8	2	9.9	1.8	Qualified
G2017-2	NC	1006.7	1.95	9.6	1.9	Qualified
G2017-3	NC	1067.7	2	9.8	2.1	Qualified
G2018-1	OE	538.1	1.97	10	2.1	Qualified
G2018-2	OE	699.4	2.03	10	2.1	Qualified
G2018-3	OE	65.2	2.01	9.9	1.9	Qualified

Quality control standard: Thermo NanoDrop 2000 (1.7< A260/A280 <2.2), Agilent 2100 Bioanalyzer (RIN>=7.0 and 28S/18S>0.7).

Compared with the control group, BM-MSCs had 522 genes with ≥1.5-fold differences in gene expression (189 upregulated, 333 downregulated) after SCD1 overexpression. Twelve genes had >3 times differential expression (2 upregulated, 10 downregulated) (Supplementary Figure 1A–1G). Classical pathway analysis suggested that these DEMs are involved primarily in important biological functions, such as activation of the nuclear factor erythroid 2–related factor 2 (NRF2)-mediated oxidative stress response pathway (Figure 2D). SCD1 overexpression downregulated inhibitory factors in this pathway, such as FOS. Disease and function analyses suggested that the DEMs are involved mainly in biological functions such as Cell Death and Survival, Cancer and Organismal Injury and Abnormalities (Figure 2E, and Supplementary Figure 1J). Bioinformatics analysis revealed that CDKN1A, with |logFC| > 1.5, plays a vital role in these pathways. However, as our subsequent experiments showed that CDKN1A was not as significant as FOS in this setting (data not shown), we did not further examine its role of CDKN1A in SCD1-overexpressing MSCs.

The first regulatory network identified in the regulation effect analysis consisted of ALB, BTC, CAMP, CXCL8, F2R, IL22, MARK2, NFATC2, PF4, PTPRJ, SAMSN1, TBK1, Tnf (family), TXN, and VCAN. These regulators have activation effects on organismal death through ANGPT1, CCL20, CDKN1A, CEBPD, COL1A1, CRISPLD2, CTGF, CXCL2, CXCL3, CYP1B1, F3, HAS1, HSPA5, IL1A, IL1R1, INHBA, NFKBIZ, PTGS2, RBL2, SAA1, SOD2, SORT1, STAT2, and TNFAIP3, and inhibitory effects on myeloid cell movement and connective tissue cell pathway development (Supplementary Figure 1H). PPI network analysis revealed connections among biomolecules in the dataset. The network map with the highest score affected mainly organ development and morphology, and reproductive system development and function, with CDKN1A playing a key role (Supplementary Figure 1I). Together, these results suggest that SCD1 regulates the NRF2-mediated oxidative stress response, mitotic roles of polo-like kinase, hepatic stellate cell activation, and hepatic fibrosis, controlling necrosis and organismal death/survival.

### DE-miRNAs and DEMs selected from GEO datasets

**Figure 3:**
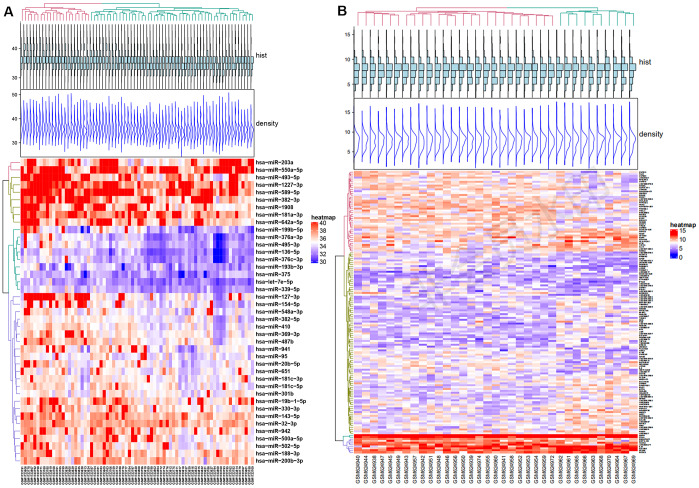
**Differential expression of miRNA and mRNA.** (**A**) Heat map of Differentially expressed micro-RNAs from GSE70318. (**B**) Heat map of Differentially expressed mRNAs from GSE25462. Red: upregulation; Blue: downregulation. GSE70318 provides information on serum miRNA signals regarding fractures in postmenopausal women with or without type 2 diabetes. GSE25462 is a dataset of miRNAs related to diabetes and skeletal muscle insulin resistance. We did not find a miRNA dataset directly related to diabetic skeletal disease; considering the close relationship between skeletal muscle and bone (especially fracture risk), and the large size and high quality of the GSE25462 dataset, we used these data.

The expression levels of genes acquired from the GSE70318 and GSE25462 datasets are shown in Figure 3A, 3B. GSE70318 provides information on serum miRNA signatures that indicate skeletal fracture and influence osteogenic differentiation of MSCs in post-menopausal women with and without T2DM. GSE25462 is a dataset of miRNAs related to diabetes and skeletal-muscle insulin resistance. We found no miRNA dataset related directly to diabetic bone disease; considering the close relationship between skeletal muscle and bone (and especially fracture risk), and the large size and good quality of the GSE25462 dataset, we used these data in this study (Supplementary Figure 2A–2D). We speculated that the biological effects of SCD1 on skeletal muscle and BM-MSCs were similar. Our experiments revealed that the overexpression of SCD1 led to the overexpression of miR-1908 and miR-203a, confirming this hypothesis. After normalization, the gene distribution was uniform and adequate for further study (Supplementary Figure 2E). Volcano plots of differential expression are presented in Supplementary Figure 2F. For GSE70318 data, hsa-miR-382-3p, hsa-miR-369-3p, hsa-miR-376c-3p, and hsa-miR-1908 were the most upregulated miRNAs and hsa-miR-550a-5p, hsa-miR-203a, and hsa-miR-942 were the most downregulated miRNAs in the diabetic fracture group. In total, 147 DEMs (80 upregulated, 67 downregulated) were obtained from the GSE25462 skeletal muscle samples. Matrix data for differentially expressed from GSE70318 and DEMs from GSE25462 are presented as heatmaps in Figure 3A, 3B.

DEMs in the GSE25462 dataset showed enrichment in BPs (drug responses, negative regulation of endopeptidase activity) MFs (serine-type endopeptidase inhibitor activity, protein heterodimerization, calcium ion binding), and CCs (negative regulation of apical plasma membrane and extracellular region; Supplementary Figures 3A and 4). Pathway analysis showed that the DEMs were involved mainly in autoimmune thyroid disease, the oxytocin signaling pathway, bile secretion, and the T cell receptor signaling pathway (*P* < 0.05; Supplementary Figure 5A, 5B). The most significantly enriched genes in the network were associated with the oxytocin signaling pathway (Supplementary Figure 5). Enriched KEGG pathways were the oxytocin signaling and Epstein−Barr virus infection pathways for upregulated DEMs, and autoimmune thyroid disease pathways for downregulated DEMs (Supplementary Figures 3B and 5). Target genes of both upregulated and downregulated DEMs were enriched for histidine metabolism.

### miRNAs selected by predictive modeling of diabetic fracture risk

**Figure 4:**
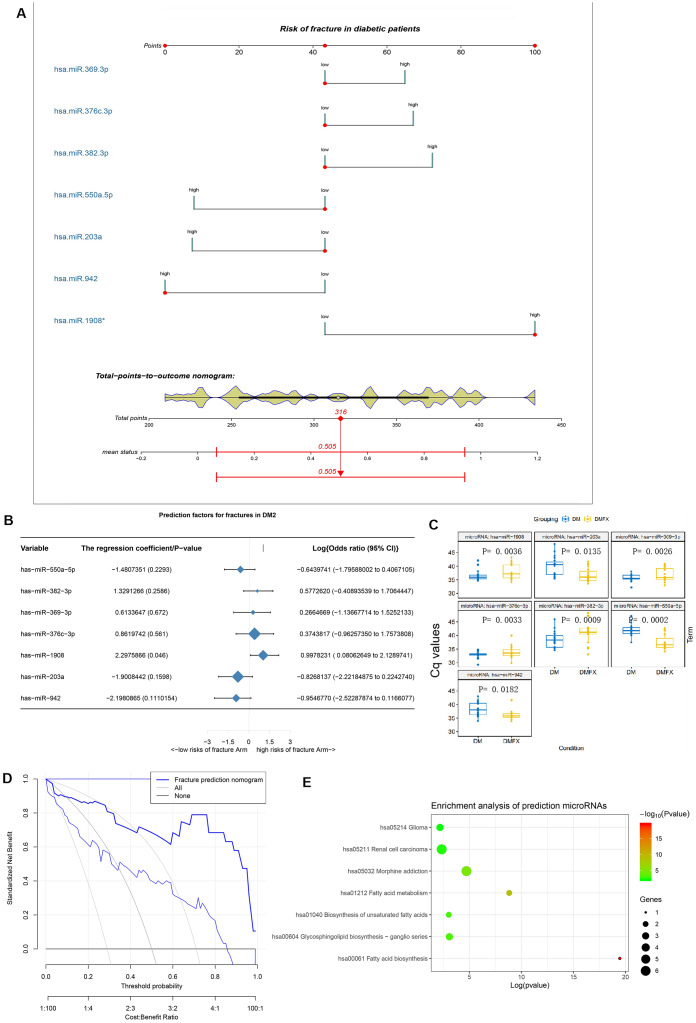
**The establishment and validation of the risk prediction model of diabetic fracture.** (**A**) Developed diabetic fracture nomogram. Note: The diabetic fracture nomogram was developed in the cohort, with hsa-miR-550a-5p, hsa-miR-382-3p, hsa-miR-369-3p, hsa-miR-376c-3p, hsa-miR-1908, hsa-miR-203a and hsa-miR-942 incorporated. (**B**) Predictive factors of fractures in patients with DM2. (**C**) Differential miRNA expression levels between with (DMFX) and without (DM) fragility fractures in the postmenopausal women with type 2 diabetes. To enable intuitive interpretation of upregulation and down-regulation, Cq values are inverted along the *y* axis. (**D**) Decision curve analysis for the diabetic fracture nomogram. (**E**) Enrichment analysis of hsa-miR-550a-5p, hsa-miR-382-3p, hsa-miR-369-3p, hsa-miR-376c-3p, hsa-miR-1908, hsa-miR-203a and hsa-miR-942 using mirPath v.3.

Based on the LASSO regression model, we selected 7 key microRNA predictors of fracture risk in diabetic patients from 10 features: hsa-miR-550a-5p, hsa-miR-382-3p, hsa-miR-369-3p, hsa-miR-376c-3p, hsa-miR-1908, hsa-miR-203a, and hsa-miR-942 (Table 2; Supplementary Figure 6A, 6B). A nomogram model incorporating these independent predictors is shown in Figure 4A–4C. The model showed strong performance in the prediction of fracture risk in patients with T2DM (Supplementary Figure 6C). The C-index of the nomogram for the test cohort was 0.934 (95% CI, 0.874–1.000), and was validated to be 0.919 by bootstrapping, suggesting strong discriminatory power and accurate predictive performance (Supplementary Figure 6D). The decision curve showed that application of the nomogram for the prediction of fracture risk is beneficial relative to the scheme without clinical interventions (Figure 4D). In this range, this predictive model can better guide clinical practice, including early intervention to manage fracture risk factors, thereby reducing this risk in patients with diabetes. Enriched KEGG pathways for all of these DE-miRNAs were the oxytocin signaling and Epstein−Barr virus infection pathways, and miR-1908 was enriched in all of noteworthy pathways (Figure 4E).

**Table 2:** DE-miRNAs' expression (from GSE70318) of DM and DMFX between all groups.

**Group**	**DE-miRNAs**	**Log FC**	**Ave Expr**	**t**	**P value**	**adj. P value**	**B**
DM vs. DF	hsa-miR-1908	1.971433518	37.25828601	3.108424187	0.003589719	0.078461002	-1.932506566
DM vs. NF	hsa-miR-1908	2.072686981	37.53576177	2.625233193	0.012484124	0.159172582	-2.862992842
DM vs. DF	hsa-miR-550a-5p	-3.297139889	39.68187327	-4.175946403	0.00017111	0.013089881	0.809395368
DM vs. NF	hsa-miR-550a-5p	-2.052091413	40.22924515	-2.559383961	0.014673316	0.17269364	-2.99553445
NM vs. DF	hsa-miR-550a-5p	-3.820107929	39.4024267	-4.64777946	4.55E-05	0.003477881	2.009361175
DM vs. DF	hsa-miR-376c-3p	1.459958449	33.26060249	3.136343478	0.003330441	0.078461002	-1.865877696
NF vs. DF	hsa-miR-376c-3p	1.088885042	33.40756579	2.528879569	0.015800129	0.117673065	-3.272405749
NM vs. DF	hsa-miR-376c-3p	1.510121259	33.03627315	3.055844618	0.00425526	0.059186792	-2.181922657
DM vs. DF	hsa-miR-382-3p	3.257160665	39.2225831	3.621318593	0.000866714	0.044202412	-0.659953251
NF vs. DF	hsa-miR-382-3p	2.358594183	39.41555055	2.392036678	0.021906492	0.139653883	-3.554802592
NM vs. DF	hsa-miR-382-3p	3.495299708	38.76599151	3.700970715	0.00072775	0.019514414	-0.567368574
DM vs. DF	hsa-miR-369-3p	1.594729917	36.12329294	3.23304419	0.002562973	0.078461002	-1.632574009
NF vs. DF	hsa-miR-369-3p	1.450477839	36.14316136	3.19582501	0.002837101	0.075890586	-1.753163692
NM vs. DF	hsa-miR-369-3p	1.977112143	35.58799383	4.04832837	0.000268212	0.013678815	0.356239113
DM vs. DF	hsa-miR-203a	-2.246267313	38.16271122	-2.592728721	0.013519407	0.188042665	-3.097298217
DM vs. NF	hsa-miR-203a	-2.686184211	38.03373615	-3.219709086	0.00265963	0.070320793	-1.575412588
DM vs. DF	hsa-miR-942	-1.531087258	37.1696295	-2.470627692	0.018176238	0.19864032	-3.352699045

Note:

DM (postmenopausal women with T2DM, without histories of at least one osteoporotic fracture),

NM (postmenopausal women without T2DM, without histories of at least one osteoporotic fracture),

NF (postmenopausal women without T2DM, with histories of at least one osteoporotic fracture),

DF (postmenopausal women with T2DM, with histories of at least one osteoporotic fracture).

Representation:

DM *vs.* DF: the DE-miRNAs between DM and DMFX.

DM *vs.* NF: the DE-miRNAs between DM and NF.

NF *vs.* DF: the DE-miRNAs between NF and DF.

NM *vs.* DM: the DE-miRNAs between NM and DM.

### miR-1908 and miR-203a levels are related to SCD1 expression

**Figure 5:**
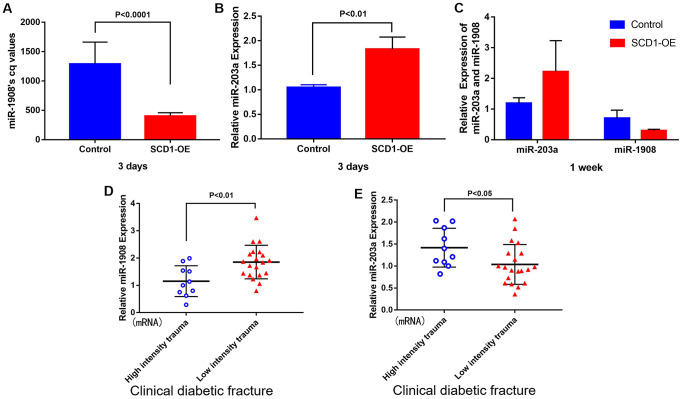
**Correlation between miR-1908 and miR-203a expression levels and SCD1.** Differential expression of miR-1908 and miR-203a after 3 days (**A**, **B**) and 1 week (**C**) of SCD1 overexpression in BM-MSCs. (**D**, **E**) showed the relative expression of miR-1908 and miR-203a detected by qPCR in patients with clinical diabetic fractures.

The levels of miR-1908 and miR-203a in the SCD1-OE group were associated significantly with the expression of SCD1, as determined by quantitative real-time PCR analysis (*P* < 0.05, Figure 5A–5C). Positive miR-1908 expression and negative miR-203a expression were each observed in patients with low-energy fracture (Figure 5D, 5E).

### PPI network and interrelationships between pathways

**Figure 6:**
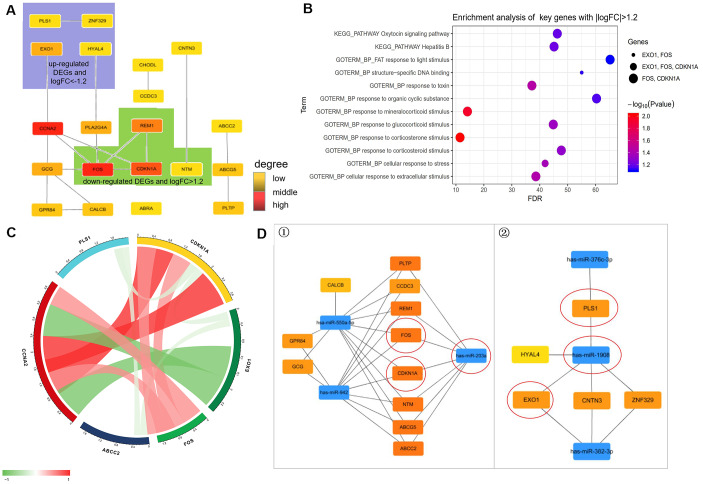
**Prediction of molecules interaction network.** (**A**) Modules inferred from protein-protein interaction (PPI) network. The degrees of connectivity are represented by different colors, with red representing strong correlation. (**B**) Enrichment analysis of key genes with |logFC|>1.2 including “HYAL4”, “ZNF329”, “PLS1”, “EXO1”, “FOS”, “GPR84”, “CALCB”, “NTM”, “CDKN1A”, and “CHODL”. (**C**) Correlation of diabetes highly related molecules (key genes with |logFC| > 1.2). (**D**) The regulatory network between dysregulated miRNAs and hub genes. ①. For downregulated miRNAs; ②. For upregulated miRNAs. Notes: “circle” means Co-Differentially expressed mRNAs after overexpression of SCD1.

In STRING network analysis, 104 nodes and 86 edges were identified in the GSE25462 data, with a PPI enrichment *P* value of 8.19e-07 (Supplementary Figure 7). Based on the PPI network, modules were identified, and 19 hub genes were screened out (Figure 6A). FOS, CDKN1A, REM1, NTM, EXO1, PLS1, ZNF329, and HYAL4 played important roles in the network (*P* < 0.05, |log FC| ≥ 1.2; Figure 6A). According to enrichment analysis, GO functions were associated mainly with responses to corticosterone, mineralocorticoid, glucocorticoid, and corticosteroid stimuli, toxins, extracellular stimuli, and stress. KEGG pathways were associated mainly with oxytocin signaling and hepatitis B (Figure 6B). FOS, CDKN1A and EXO1 were involved in the most enrichment processes, with high degrees of interaction (Table 3).

**Table 3:** Key DEMs with logFC>1.2.

**DEMs of GSE25462**	**Log FC**	**Average expression**	**t**	**P value**	**Adjusted P value**	**B**	**Combined score of string**
HYAL4	-1.36517	8.247334	-2.73949	0.009551	0.476416	-2.61867	<0.9
ZNF329	-1.36517	8.247334	-2.73949	0.009551	0.476416	-2.61867	<0.9
PLS1	-1.35695	8.201552	-3.10767	0.003693	0.4401	-1.89868	<0.9
EXO1	-1.32507	8.755316	-4.02141	0.000287	0.3128	0.051724	≥0.9
FOS	1.211449	6.710579	2.256556	0.030271	0.587803	-3.47818	≥0.9
GPR84	1.31698	7.330985	2.425427	0.02049	0.556097	-3.19002	≥0.9
CALCB	1.336993	7.669626	3.199253	0.002892	0.426542	-1.71246	≥0.9
NTM	1.501358	8.30572	3.881903	0.00043	0.342329	-0.25681	≥0.9
CDKN1A	1.554932	8.23666	3.865263	0.000451	0.342329	-0.29342	≥0.9
CHODL	1.554932	8.23666	3.865263	0.000451	0.342329	-0.29342	<0.9

Pearson correlation analysis showed that CDKN1A correlated significantly with molecules associated with its function, including FOS (*r* D= 0.518), PLS1 (*r* D = –0.224), and EXO1 (*r* D = –0.203). CCNA2 also correlated with molecules associated with its function, including CDKN1A (*r* D = 0.999), FOS (*r* D = 0.535), and EXO1 (*r* D = –0.87; Figure 6C).

Based on the miRWalk3.0 and miRTarBase databases, linkage between DEMs and miRNAs is displayed in Figure 6D. Ten hub genes (ABCG5, PLTP, CCDC3, REM1, FOS, CDKN1A, NTM, ABCC2, GCG, and CALCB) were likely influenced by the down-regulation of miR-550a-5p in T2DM. Three hub genes (EXO1, CNTN3, and ZNF329) were modulated by miR-382-3p. And miR-1908 targeted five hub genes (HYAL-4, PLS1, EOX1, CNTN3, and ZNF329) in the diabetic fracture group. Ten hub genes were controlled by miR-942. Combined with the logistic regression results, these results indicate that miR-203a and miR-1908 are key modulators of fracture and nonunion in patients with diabetes. FOS and CDKN1A were the strongest targets of hsa-miR-203a, and PLS1 and EXO1 appeared to be the strongest targets of hsa-miR-1908. hsa-miR-203a and hsa-miR-1908 displayed similar expression patterns.

### CDKN1A, FOS, EXO1 and PLS1 are regulated by SCD1

**Figure 7:**
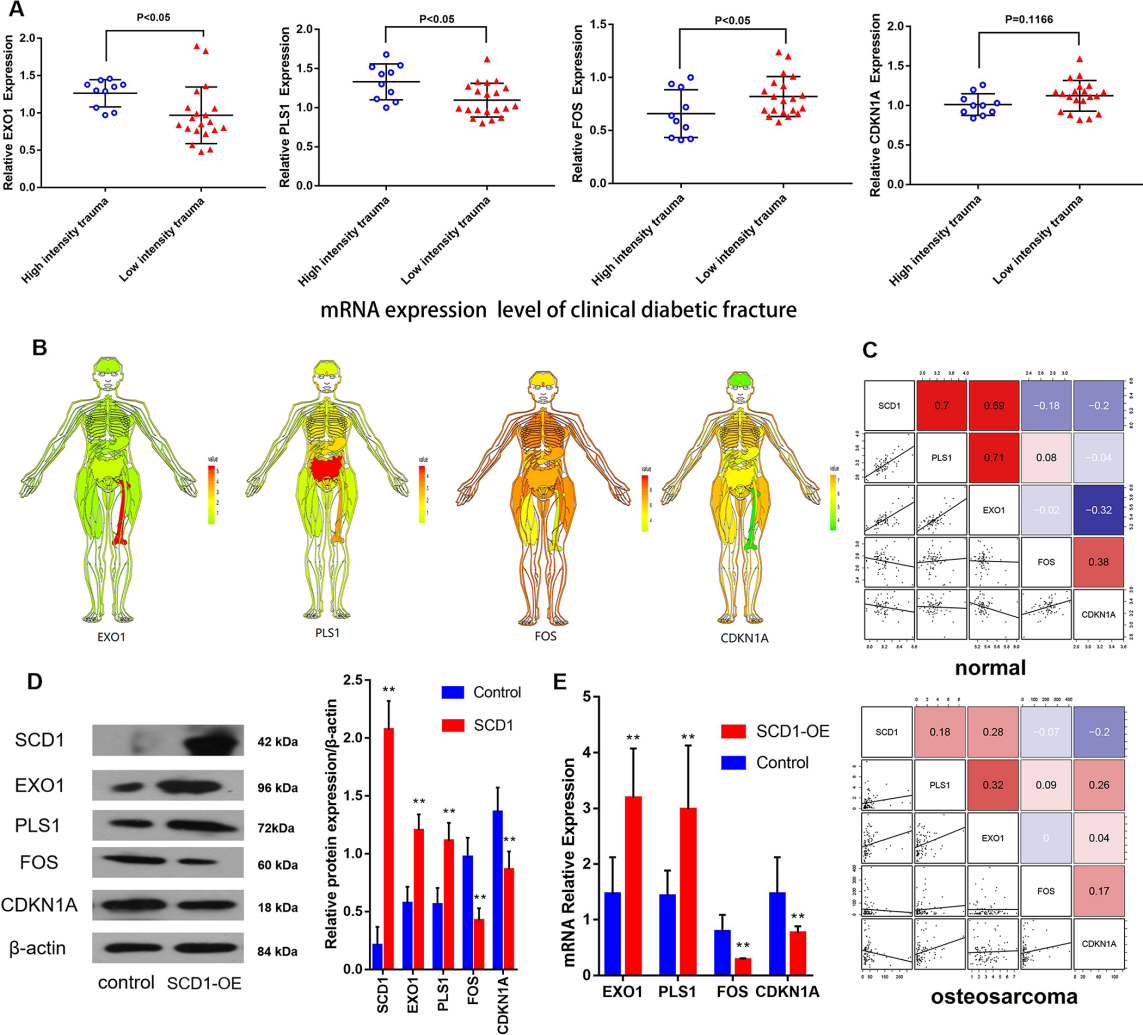
**CDKN1A, FOS, EXO1 and PLS1 are regulated by SCD1.** (**A**) Independent *t*-test results for the association between mRNAs (EXO1, PLS1, FOS and CDKN1A) and trauma energy in patients with clinical diabetic fractures patients. (**B**) Human tissue-enriched protein expression map of EXO1, PLS1, CDKN1A and FOS. (**C**) In the bone marrow, PLS1 and EXO1 are highly expressed, whereas CDKN1A and FOS are relatively low. (**D**) A represent Western blot showing overexpression of SCD1 in BM-MSCs transduced with lentivirus (“SCD1-OE”). “Control” cells are intact BM-MSCs before transduced with lentivirus. (**E**) Relative expression of mRNAs (EXO1, PLS1 FOS and CDKN1A) showing overexpression of SCD1 in BM-MSCs transduced with lentivirus (“SCD1-OE”); “Control” cells are intact BM-MSCs before transduced with lentivirus.

Based on the above-reported findings, hsa-miR-203a and hsa-miR-1908 were selected as co–DE-miRNAs of GSE70318 and BM-MSCs after SCD1 overexpression. PLS1, EXO1, FOS, and CDKN1A were selected as co-DEMs of GSE25462 and BM-MSCs after SCD1 overexpression. Quantitative real-time PCR showed that the average expression levels of EXO1 and PLS1 were significantly lower in the bone marrow of diabetic patients with low-energy fracture than in that of controls, whereas the opposite was observed for FOS and CDKN1A (Figure 7A). To understand the expression of these genes in the human body more intuitively, we constructed human tissue–enriched protein expression maps. We found that SCD1, PLS1, and EXO1 are highly enriched in bone, whereas CDKN1A and FOS expression levels in bone are relatively low (Figure 7B, 7C). The expression of osteogenesis-promoting proteins may be higher and that of osteogenesis-free proteins may be lower in the normal bone environment; thus, SCD1, EXO1, and PLS1 may be more important for osteogenesis, whereas FOS and CDKN1A could be detrimental to skeletal development. Furthermore, gene microarray assays, western blotting and qPCR confirmed that SCD1 inhibits CDKN1A/FOS expression, and may upregulate EXO1/PLS1 (Figure 7D, 7E).

### miR-1908 inhibits and miR-203a promotes the proliferation and osteogenic differentiation of BM-MSCs

**Figure 8:**
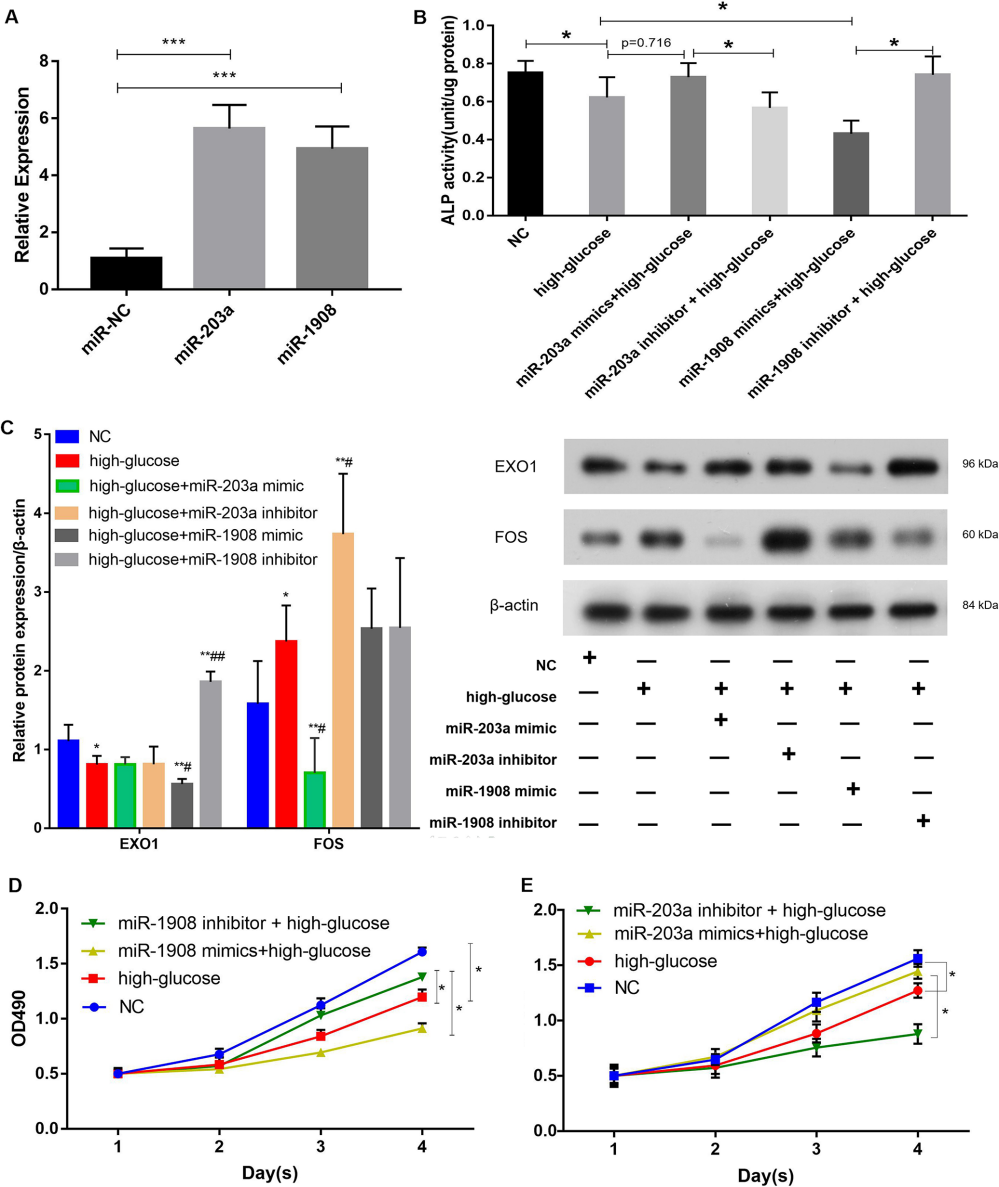
**miR-1908 inhibits and miR-203a promotes the proliferation and osteogenic differentiation of BM-MSCs.** (**A**) Transfection efficiency of miR-203a and miR-1908 in BM-MSCs. (**B**) ALP activity was measured in BM-MSCs treated with miRNA inhibitor or mimic (miR-203a and miR1908). (**C**) The expression of EXO1 and FOS were assessed in BM-MSCs transfected with miR-203a mimic, miR-203a inhibitor, miR-1908 mimic and miR-1908 inhibitor. (**D**, **E**) Proliferation of BM-MSCs following the evaluation of the overexpression and knockdown of microRNAs (miR-203a and miR1908).

We next aimed to determine whether miR-1908 and miR-203a regulated the osteogenic differentiation of BM-MSCs. First, the expression of miR-203a and miR-1908 was greatly enhanced by infection with mimics (Figure 8A), and an ALP assay showed that the miR-1908 mimic reduced ALP activity, whereas the miR-203a mimic increased it (Figure 8B). Then, the protein expression of the BM-MSCs was analyzed by western blotting under the condition of infected with an miR-203a or miR-1908 mimic or transfected with an miR-203a or miR-1908 inhibitor respectively (Figure 8C). The miR-203a mimic significantly reduced the expression of FOS, and the miR-1908 mimic significantly decreased the expression of EXO1. The proliferation of BM-MSCs after miRNA overexpression or knockdown was then evaluated by MTT assay. The miR-1908 mimic significantly reduced proliferation at 2, 3, and 4 days, whereas the miR-203a mimic increased proliferation at the same timepoints (Figure 8D, 8E). These data indicate that miR-1908 reduces the proliferation and differentiation of BM-MSCs, whereas miR-203a increases them, in a hyperglycemic microenvironment.

### Identification of miR-1908 and miR-203a as direct regulators of EXO1 and FOS expression, respectively, in the hyperglycemic microenvironment

**Figure 9:**
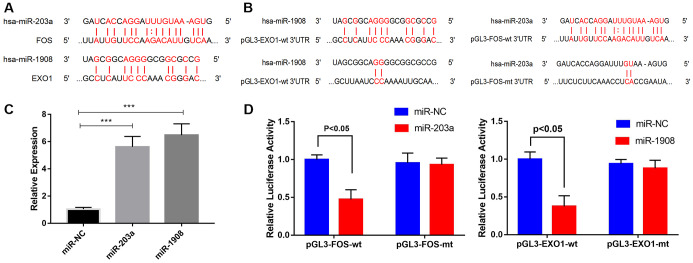
**Prediction and verification of EXO1 and Fos expression regulated directly regulation by miR-1908 and miR-203a respectively.** (**A**) Putative miR-203a and miR-1908 binding sites in the 3'-untranslated regions of FOS mRNA (predicted by miRanda) and EXO1 mRNA (predicted by miRWalk), respectively. (**B**) Luciferase reporter assays to evaluate FOS regulation by miR-203a and EXO1 regulation by miR-1908. (**C**) Transfection efficiency of miR-203a and miR-1908 in UM-Chor1 cells under the hyperglycemic circumstance. (**D**) UM-Chor1 cells co-transfected with pGL3-FOS-wt and miR-203a vs. those co-transfected with pGL3-FOS-mt and miR-NC; UM-Chor1 cells co-transfected with pGL3-EXO1-wt and miR-1908 vs. those co-transfected with pGL3-EXO1-mt and miR-NC.

The MiRWalk and miRanda databases predict that EXO1 and FOS are regulated by hsa-miR-1908 and hsa-miR-203a, respectively (Figure 9A). We performed luciferase reporter assays to determine whether miR-203a regulated FOS expression and miR-1908 regulated EXO1 expression (Figure 9B). We confirmed the transfection efficiency of miR-1908 and miR-203a in UM-Chor1 cells (Figure 9C). UM-Chor1 cells co-transfected with pGL3-FOS-wt and miR-203a showed less luciferase activity than did those co-transfected with pGL3-FOS-mt and miR-negative control (NC; *p* < 0.05). Similarly, UM-Chor1 cells co-transfected with pGL3-EXO1-wt and miR-1908 showed less luciferase activity than did those co-transfected with pGL3-EXO1-mt and miR-NC (*P* < 0.05, Figure 9D). These results support the bioinformatics predictions and indicated that miR-1908 and miR-203a were direct regulators of EXO1 and PLS1 expression, respectively, in bone marrow.

### miR-203a/FOS and miR-1908/EXO1 are regulated by SCD1

**Figure 10:**
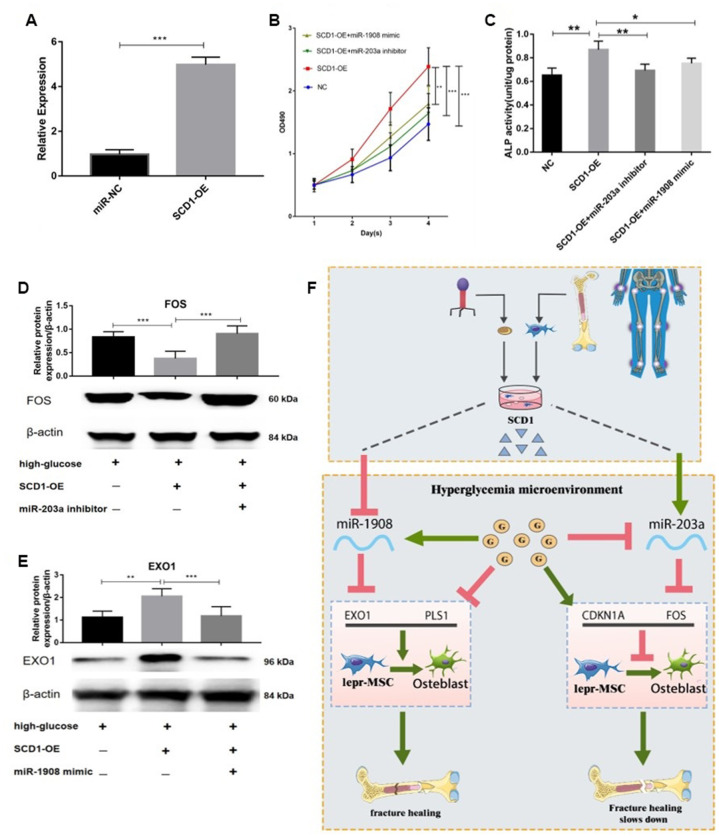
**miR-203a/FOS and miR-1908/EXO1 are regulated by SCD1.** (**A**) Transfection efficiency of SCD1 in BM-MSCs. (**B**) Proliferation of BM-MSCs after SCD1 overexpression, miR-1908mimic and miR-203a inhibitor were evaluated. (**C**) ALP activity was measured in SCD1-overexpressing BM-MSCs treated with miR-203a inhibitor or miR-1908 mimic. (**D**, **E**) Expressions of FOS and EXO1 were assessed in BM-MSCs transfected with high-glucose, SCD-OE miR-1908 mimic and miR-203a inhibitor. (**F**) The regulatory network between dysregulated miRNAs and hub genes after overexpression SCD-1.

First we confirmed the transfection efficiency of SCD1 in BM-MSC cells (Figure 10A). The proliferation of BM-MSCs was then evaluated after overexpression of SCD1 and co-transfection with the miR-203a inhibitor or miR-1908 mimic. Cells transfected with SCD-1 showed significantly increased proliferation compared with the others, whereas proliferation was decreased in cells treated with the miR-203a inhibitor and miR-1908 mimic (Figure 10B). Similar effects were observed for ALP activity; miR-1908 markedly reduced the ALP level, whereas miR-203a played a protective role (Figure 10C). Western blots showed significantly reduced FOS expression and increased EXO1 expression in SCD1-overexpressing cells, whereas the expression of FOS was increased by treatment with the miR-203a inhibitor, and the combination of SCD1-OE and miR-1908 mimic resulted reduced the expression of EXO1 to control levels (Figure 10D, 10E). Therefore, MiR-203a/FOS pathway may be repressed and the MiR-1908/EXO1 pathway could be active in patients with diabetic fracture (Figure 10F). SCD1 may protect bone from fracture in diabetic patients by regulating miRNA expression changes caused by diabetes, such as those in hsa-miR-203a and hsa-miR-1908.

## DISCUSSION

The present study revealed that SCD1 is involved in the regulation of hsa-miR-203a and hsa-miR-1908, which mediate the expression of FOS and EXO1 and may be associated with diabetic fracture. Additionally, we developed a novel predictive tool for fracture risk in patients with T2DM using seven available variables. Importantly, the results of this research suggest that six crucial miRNAs (hsa-miR-1908, hsa-miR-943, hsa-miR-203a, hsa-miR-550a-5p, hsa-miR-382-3p, and hsa-miR-376c-3p) regulate the expression of 16 genes (especially in FOS, PLS1, EXO1, and CDKN1A), thereby influencing how T2DM exerts negative effects on bone.

Gene microarray analysis revealed that SCD1 downregulated the expression of CDKN1A and FOS, and possibly upregulated the expression of EXO1 / PLS1. The miRNA-mRNA interaction network predicted that EXO1 / PLS1 and CDKN1A / FOS expression is regulated by hsa-miR-1908 and hsa-miR-203a, respectively. Western blot analysis further showed that CDKN1A and FOS expression was significantly reduced, whereas EXO1 and PLS1 expression was increased, in BM-MSCs transfected with SCD1. These results indicate that SCD1 inhibited the expression of CDKN1A and FOS by promoting hsa-miR-203a. Hsa-miR-1908 might also be silenced by SCD1 and promote the expression of EXO1 and PLS1. However, further research is needed to reveal the positive effects of SCD1 on diabetic fractures.

SCD1 modulates the biological metabolism of cells and promotes the formation of unsaturated fatty acids. [21, 36] Our study showed that SCD1 overexpression promotes BM-MSC osteogenesis *in vitro* in the context of diabetic bone disease, similar to previous findings [18]. In addition, SCD1 overexpression significantly downregulated the activity of NRF2 pathway–associated genes. This pathway is associated with antioxidants and is activated by increases in oxygen free radicals in the body [37]. Park et al. [38] suggested that Nrf2 promotes antioxidant synthesis to bind oxygen free radicals and repair cellular damage caused by them. Loss-of-function mutation of Nrf2 decreased load-driven anabolic responses and bone mass [39], indicating that Nrf2 has important regulatory functions in bone homeostasis. In this study, despite NRF2 pathway down-regulation, osteogenesis-related gene expression was stronger in the SCD1-overexpressing group than in the control group. On the other hand, our disease and functional analyses showed significant upregulation of genes involved in cell differentiation and proliferation with SCD1 overexpression. Some previous studies have shown that SCD1 promotes the proliferation and survival of cells [40–42]. In addition, cell cycle regulation depends on the synthesis of unsaturated fatty acids, and SCD1 affects the cell cycle by regulating the levels of cyclin D1 and cyclin-dependent kinase 6, which play important roles in the G1/S phase [43]. SCD1 may promote bone formation by regulating osteoblast differentiation and proliferation.

In this study, we observed reduced levels of hsa-miR-203a in diabetic fracture samples. We did not know if this reduction was related to poor glycemic control, as miR-203 expression in diabetic patients is downregulated at all timepoints [44, 45]. Modification of the miR-203 level in diabetic mice resulted in marked apoptosis of beta cells [46]. Based on our diabetic fracture nomogram, we postulated that high miR-203 expression promotes MSC differentiation into osteoblasts and helps to reduce fracture risk, and this result was similar to previous research results [47]. Similarly, miR-203 promotes stem cell differentiation by inducing cell cycle exit via its target protein p63 [48]. Pre-transduction of miR-203 into BM-MSCs alleviated cell injury induced by low nutrition, which enhanced BM-MSC survival [49]. Alternatively, high miR-203a expression may prevent fracture and bone damage by blocking the nuclear factor (NF)-κB signaling pathway, but this possibility requires further investigation.

Our bioinformatics analysis suggested that SCD1 induced CDKN1A down-regulation. The effects of CDKN1A are thought to play vital roles in pathways related to cell death and survival. CDKN1A/P21, a cyclin-dependent kinase inhibitor, plays an important role not only in cell proliferation and differentiation, but also in apoptosis [50, 53]. CDKN1A knockdown in late-passage MSCs increased the cells’ osteogenic and proliferation capacities [51]. CDKN1A expression in mesenchymal progenitor cells differs between osteoarthritic and non-arthritic individuals, with high expression correlated with reduced chondrogenic potential [52].

FOS, a member of the AP-1 family of transcription factors, participates in osteogenesis [54, 55]; according to our findings, it is regulated by miR-203a. The hyperglycemic microenvironment increases c-Fos expression, impairing mitochondrial function and osteoblast differentiation in the bone microenvironment [56, 57]. In diabetic animal models, curcumin and Zinc inhibited bone resorption by limiting osteoclastogenesis and the expression of c-Fos or reduced receptor activator of nuclear factor-κB ligand expression a fos-related pathway [58–60]. Some anti-diabetic drugs, such as sitagliptin and thiazolidinediones, also promote osteogenesis through anti-Fos [61, 62]. This evidence suggests that anti-FOS treatment benefits bone health in diabetic patients. Our findings may provide the basis for new ideas about the treatment of diabetic osteopathy.

miR-1908, an intragenic miRNA, is located in the first intron of the FADS1 gene on human chromosome 11. Clinical studies have shown that patients with high FADS1 expression are more susceptible to metabolic syndrome and inflammatory reactions in response to dietary linoleic acid [62, 63]. miR-1908 expression is also increased in human mature adipocytes, and this miRNA is involved in the regulation of Wnt receptor signaling, glucose/insulin metabolism, the cell cycle, and cell apoptosis; it thus regulates the differentiation and proliferation of normal adipocytes and multiple cytokines [64, 65]. In our study, we observed upregulated hsa-miR-1908 expression in diabetic fracture samples. Jiang et al. [65] also reported the correlation between TNF-α and miR-1908. TNF-α, as the activator of NF-κB, is upregulated in diabetic patients [66–68]. As a transcriptional factor, NF-κB fine tunes inflammatory responses and promotes bone loss through effects on osteoclasts and osteoblasts [69, 70]. MiR-1908 expression functions are enforced by positive regulation of the NF-κB pathway [71, 72]. Therefore, miR-1908, which was upregulated in diabetic fracture samples in our study, may be associated with an NF-κB/TNF-α–related pathway and osteoblast apoptosis, but more studies needed to be conducted.

In addition, EXO1 and PLS1, which are regulated by SCD1, are related to diabetic fracture development. EXO1 and PLS1 were downregulated in our T2DM samples. Some types of actin-mediated hearing impairment are influenced by mutations in the human PLS1 gene [73]. Plastins contribute, in an isoform-specific manner, to the development of invadosomes of immune and cancer cells, adhesion contacts, endocytic patches, immune synapses, inner ear stereocilia, and intestinal and kidney microvilli [73–76]. Thus, PLS1, an essential part of actin that was downregulated in our T2DM samples, may be linked to the function or growth of skeletal muscle in patients with T2DM. Additional studies of the effects of PLS1 on skeletal muscle and T2DM are needed.

EXO1, which this study showed is targeted by miR-1908, maintains genome stability by regulating telomeres and DNA replication and recombination [77, 78]. EXO1 expression was higher in 395 German centenarians than in 411 controls, showing the importance of this function in increasing longevity in humans [79]. A genome-wide association study showed that a lack of EXO1 contributed to low BMD [80], suggesting that EXO1 holds huge promise as a marker for the early detection of osteoporotic fracture.

This study is the first to detail the serum profiles of miRNAs and mRNAs in women with T2DM and their effects on fracture risk, and to suggest that SCD1 could be beneficial in the treatment of diabetic patients at high risk of fracture. It demonstrates that miRNA signatures can be used to study miRNA–mRNA interactions in in vitro experiments. We anticipate that additional experiments will leverage the novel findings presented here to expand our knowledge about the roles of differentially expressed mRNAs and miRNAs in bone tissue and to develop clinically important molecular markers for fracture risk assessment in the context of diabetes. The major strength of this study is that we constructed a PPI network coupled with an SCD1–miRNA–mRNA interaction network for T2DM based on experiments. Furthermore, the nomogram developed in this study displayed a high degree of accuracy and is suitable for the clinical assessment of fracture risk in patients with diabetes. This tool will help patients and clinicians to develop management and preventive measures, such as lifestyle adjustments and other suitable interventions.

The effects of the selected genes on bone health in patients with T2DM need to be verified in further experiments, and more combined bioinformatics and laboratory analyses are needed to confirm the functional implications of the identified genes. Related animal studies and additional in vitro experiments involving targeted SCD1 inhibition are also needed. This study has several limitations: (1) only the most significantly differentially expressed miRNAs and genes were included in the analysis and miRNA–mRNA interaction network construction; (2) the model does not incorporate all factors related to fracture risk in diabetic patients; (3) although bootstrap testing was used to assess the robustness of the nomogram, we did not perform external validation and thus the findings may not be generalizable to diabetic populations in other countries and regions; (4) the sample used to develop the nomogram did not include men with diabetes; (5) immunohistochemistry of bone marrow tissue in our study did not perform as well as we expected. Therefore, we suggest that further studies be performed to evaluate these findings in the context of a wider diabetic population.

In summary, the newly proposed SCD1/miR-203a/FOS and SCD1/miR-1908/EXO1 pathways may enhance our understanding of the patho-mechanisms of osteogenesis in diabetes. The findings may aid the early detection and prevention of bone disease induced by T2DM. In addition, this study provides new targets and a nomogram prediction model that form the foundation for further exploration of the molecular causes, biomarkers, and treatments of fracture in the context of T2DM.

## MATERIALS AND METHODS

### Study participants and sample collection

This study involved a total of 30 postmenopausal women with diabetes aged 55–70 years, comprising 20 women with low-energy lower-limb fracture and 10 otherwise healthy premenopausal women with high-energy lower-limb fracture, who were recruited at Shanghai General Hospital between August 2017 and January 2019 (Tables 4, and 5). Human BM-MSCs were provided by Shanghai First People's Hospital from healthy postmenopausal women, to limit the effects of hormones on the outcomes. Exclusion criteria including: history of osteoporosis treatment or hormone replacement therapy, early menopause (before the age of 40 years), chronic renal failure, abnormal menopause, and acute gastrointestinal tract inflammation. In addition, healthy postmenopausal women with normal bone mineral density (BMD) and no history or X-ray evidence of fracture were recruited from the hospital’s personnel. The study complied with the Declaration of Helsinki and was approved by Shanghai First People’s Hospital (no. 2018KY094); all subjects provided written informed consent.

**Table 4:** Clinical characteristics of participants.

	**High-energy fracture**	**Low-energy fracture**
	**(n=10)**	**(n=20)**
**Age (year)**	63.6±2.1	61.9±4.3
**Length of Stay (day)**	15±4.4	9.7±3.6
**Height (cm)**	152.9±6.9	158.6±12.0
**Weight (kg)**	57.9±8.1	60.7±9.3
**BMI (kg/m^2^)**	24.8±3.3	24.1±3.4
**FBG (mmol/L)**	6.21±1.4	6.23±2.0
**2HPG (mmol/L)**	7.38±1.8	9.06±3.2
**Fracture site**		
femur	4	4
tibia	6	4
fibula	3	4
patella	0	8
calcaneus	0	1

**Table 5:** Clinical characteristics of participants grouped by the degree of blood glucose control.

**Blood glucose**	**Control well**	**Control bad**
	**(n=13)**	**(n=17)**
Age (year)	61.4±3.4	63.3±3.9
Length of Stay (day)	12.8±5.4	10.4±3.7
Height (cm)	158.6±11.7	155.2±10.1
Weight (kg)	57.6±10.8	61.4±7.1
BMI (kg/m^2^)	22.7±2.5	25.6±3.4
Low energy fracture (%)	61.5%	70.6%
Fracture site		
femur	4	4
tibia	3	7
fibula	4	3
patella	4	4
calcaneus	0	1

Note:

Blood glucose control well: HbA1c<7.0%;

Blood glucose control bad: HbA1c≥7.1%.

BM-MSCs were isolated as reported previously [81, 82]. In brief, bone marrow samples were aspirated from patients with fractures during surgery. The mononuclear cell fraction was recovered by density centrifugation over a Ficoll gradient at 800 × *g* for 20 min and washed twice with phosphate-buffered saline.

### Lentivirus transfection

A lentiviral system (with pCDH-SCD1, psPAX2, and pMD) that we previously constructed successfully was used in this study [83]; system development is described in detail elsewhere [17]. Briefly, BM-MSCs in the logarithmic growth stage were digested by trypsin, and cell suspensions were prepared at concentrations of 3–5 × 10^4^/mL and inoculated in 6-cm culture dishes; infection was carried out when the concentration of planks reached ~15–30%. The original medium was replaced with infection medium (Polybrene, #abs42025397; Absin, China), and 4 mL virus supernatant was added. Forty-eight hours after infection, green fluorescent protein–positive cells were detected by flow cytometry.

### Cell culture and treatment

Human adipose-derived MSCs (PCS-500-011) were purchased from the American Type Culture Collection (Manassas, VA, USA). After thawing at 37°C, the cells were cultured conventionally in high-glucose Dulbecco’s modified Eagle’s medium (Gibco cell culture; Thermo Fisher Scientific, Waltham, MA, USA) supplemented with 15% fetal bovine serum and 1% antibiotics (Sigma-Aldrich, St Louis, MO, USA), and incubated at 37°C in 5% CO_2_. The culture medium was replaced every 24 h throughout the experimental period.

Two study groups were formed. In the experimental group, BM-MSCs were transduced with lentiviral vector pCDH-SCD1 to form SCD1-overexpressing cells. In the empty vector (EV) group, BM-MSCs were transduced with lentivirus/empty pCDH. Downstream assays (described below) were performed at 3, 7, and 14 days to assess BM-MSC osteogenesis.

### Cell viability and proliferation

Cell viability and proliferation were measured using the Cell Proliferation Reagent Kit I (MTT; Roche, Basel, Switzerland) according to the manufacturer’s instructions and as described previously [19]. First, the stably transfected cells were digested with trypsin. The cells were then centrifuged, collected, resuspended in a single cell suspension, and seeded in 96-well plates. The cell culture was maintained in maintenance. The OD value at a wavelength of 490 nm was measured using a multifunctional microplate reader. The cell growth curve was plotted with time serving as the abscissa and OD as the ordinate. Each experiment was performed six times, and average values were used.

### Detection of SCD1 expression and activity

Total RNA was extracted from transduced BM-MSCs using Trizol (Invitrogen, Carlsbad, CA, USA). RNA integrity was confirmed by agarose gel electrophoresis, and RNA concentration and purity were evaluated by spectrophotometry (Nanodrop 2000; Thermo Fisher Scientific). Real-time polymerase chain reaction (RT-PCR) was performed using the SYBR premix Ex Taq II kit (TaKaRa Bio Inc., Shiga, Japan) with appropriate primers and the ABI Prism 7500 HT system (Applied Biosystems, Foster City, CA, USA), and detection was performed using a Bio-Rad (Hercules, CA, USA) sequence detection system. SCD1 activity was assessed by measuring the conversion of C14 stearic acid to C14 OA [84, 85]. The experiment was repeated three times.

Total RNA (1 μg) was used to synthesize cDNA with random primers and a PrimeScript RT reagent kit (TaKaRa Bio Inc.). PCR conditions were pre-denaturation at 95°C for 10 min, 40 cycles of denaturation at 95°C for 30 s and annealing at 60°C for 20 s, and extension at 72°C for 30 s. The SCD1 gene was amplified using the following primers: forward, 5’-AAAGAGAAGGGCGGAAAGC-3’ and reverse, 5’-GGCGTGATGGTAGTTGTGG-3’. GAPDH was used as an endogenous control and amplified using the forward 5’-TGACTTCAACAGCGACACCCA-3’ and reverse 5’-CACCCTGTTGCTGTAGCCAAA-3’ primers.

### Alkaline phosphatase assay and staining

The alkaline phosphatase (ALP) assay and staining were performed at the end of the appropriate culture period. The medium was removed, and the cells were washed with phosphate-buffered saline and fixed with 100% ethanol for 10 min. The ALP staining kit was acquired from the Blood Institute of the Chinese Academy of Medical Sciences (Tianjin, China). The stained cells were examined and photographed under a light microscope. Each experiment was performed six times, and average values were used.

### Microarray detection and statistical analysis

aRNA was prepared after the analysis of total RNA samples using the Nanodrop 2000 (Thermo Fisher Scientific) and Agilent 2100 devices (Agilent Technologies Inc., California, USA). According to the Affymetrix gene expression profile chip manipulation manual, cDNA, a double-stranded DNA template, and biotin-labeled aRNA were synthesized, and the aRNA was purified, then fragmented and hybridized with the chip probe. After hybridization, the chip was washed and then obtain an image and raw data. The chip scan image was analyzed using Agilent image extraction software for dot matrix analysis. GeneSpring GX software (version 11.5; Agilent Technologies Inc.) was used to homogenize the probe fluorescence intensity data for determination of differential gene expression and selection of genes with absolute expression differences > 1.5. Chip data were subjected to intelligent analysis using IPA software (http://www.ingenuity.com), including classical pathway analysis and disease and functional analyses. Regulation effect analysis was performed to determine the downstream roles and upstream regulatory networks involved in differential gene expression. All gene chip data were uploaded to the Gene Expression Omnibus (GEO) database of the National Center for Biotechnology Information (no. GSE106596).

### Data retrieval

The dataset supporting the conclusions of this article is available in the GEO database (http://www.ncbi.nlm.nih.gov/geo/). Initially, datasets in which mRNA expression was compared between muscle from humans with T2DM and that from normoglycemic insulin-resistant subjects were included, as well as those that compared miRNA from patients with and without histories of osteoporotic fracture. The abstracts and titles of studies identified were scrutinized, and the full texts of studies that met the criteria were read and evaluated. The R language affyPLM package (http://www.r-project.org/) was used to assess raw data quality (Supplementary Figure 2A–2D). [86, 87] The GSE25462 gene expression array dataset, based on the GPL20631 platform (Custom LNA™ Universal RT microRNA PCR panels), was selected for further study due to high data quality. Four groups from the GSE70318 miRNA expression array dataset were included: samples from postmenopausal women without T2DM, with (*n* = 19) and without (*n* = 16) histories of at least one (non-recent) osteoporotic fracture, and those from postmenopausal women with T2DM with (*n* = 19) and without (*n* = 19) fracture histories. All original files and platform probe annotation information files were saved.

### Identification of differentially expressed genes

All data were normalized using the “normalize between array” function of the “LIMMA” R package from the bioconductor project [88]. This package was also used to identify DEMs between diseased and normal samples from the GSE25462, and GSE70318 datasets. Thresholds set at *P* < 0.05 and |logFC| > 1. In total and 35 samples from GSE25462 (10 diabetes, 25 normal) were divided into two groups, and 83 samples from GSE70318 were divided into four groups (according to microarray data). All DEM results were saved in text format for subsequent hierarchical clustering analysis using the Complex Heatmap package.

### Logistic regression of diabetes fracture data

The series matrix from the GSE25462 dataset containing diabetes fracture miRNA information was downloaded from the GEO database. Data from patients in the DM and NM groups, including age, sex, ethnicity, and disease characteristics, were analyzed using R software (version 3.5.2; https://www.R-project.org). Our clinical data were used to confirm the resulting model.

To identify relevant risk factors in diabetic patients (*n* = 38), the least absolute shrinkage and selection operator (LASSO) method was applied and features with nonzero coefficients were selected. This method is used widely to reduce high-dimensional data [89–91]. A predictive model including the selected features was established using multivariable logistic regression (two-sided *P* < 0.05) [92]. Odds ratios with 95% confidence intervals (CIs) were calculated.

A predictive model was also established for predicting diabetic fracture risk based on all potential predictors [93, 94]. For determination of the diabetic fracture nomogram, we established calibration curves. Calibration accuracy was assessed statistically using the rms package, with high significance indicating that the model could not provide accurate calibration [95]. Harrell’s C-index was calculated to assess the discriminatory performance of the diabetic fracture nomogram and corrected by bootstrapping (1,000,000 bootstrap resampling) [95]. The clinical utility of the nomogram was assessed by decision curve analysis, by determining net benefits at various threshold probabilities in the diabetic fracture cohort [96]. The net benefit was calculated by subtracting the number of patients with false-positive results from the number of those with true-positive results, and by evaluating the negative effects of intervention nonuse relative to those of unnecessary intervention use [97].

### DEMs ontology and pathway enrichment analysis

To examine pathway enrichment and functional annotation for the predicted targets of screened DEMs, the database for annotation, visualization, and integrated discovery (DAVID 6.8; http://david-d.ncifcrf.gov/) was applied, including Kyoto Encyclopedia of Genes and Genomes (KEGG) and gene ontology (GO) pathway analyses [98]. Three categories of GO functional annotation were examined: molecular functions (MFs), cellular components (CCs), and biological processes (BPs). Values with *P* < 0.05 were saved in text format. Enrichr (http://amp.pharm.mssm.edu/Enrichr/enrich) and the R language GOplot package were used to analyze pathway enrichment (*P* < 0.10). In addition, we also examined the enrichment of differentially expressed miRNAs in GSE70318 by using mirPath v.3 (http://snf-515788.vm.okeanos.grnet.gr/).

### Protein–protein interaction network construction and module analysis

DEMs were loaded onto the Search Tool for the Retrieval of Interacting Genes (STRING; https://string-db.org/) to identify functional interactions between proteins of target genes, and the Cytoscape software (version 3.7.0, MCODE plug-in) was used to visualize interaction scores with values of 0.4 [99, 100]. Hub genes were identified by analyzing the degree of connectivity within the protein–protein interaction (PPI) network using Cytoscape. The results were used to design a miRNA–mRNA and hub gene network. The DAVID and Enrichr platforms were used for functional enrichment analysis by module. In addition, we loaded genes from each module onto Enrichr for KEGG pathway enrichment analysis using a threshold of *P* < 0.05.

PPI networks were analyzed using IPA software (http://www.ingenuity.com), which utilizes an algorithm to create segmentation on the network map between biomolecules, producing multi-networks. Scoring was performed according to the hypergeometric network distribution, and the networks were filtered based on scores. Correlations of diabetes-related molecules were examined, and a ternary plot of gene expression frequencies was generated, using the “circlize” and “ggtern” packages, respectively.

### Prediction of miRNA targets

Differentially expressed mRNAs (DEMs) were obtained using the parallel method described above. The miRWalk3.0 database (http://mirwalk.umm.uni-heidelberg.de/), which includes 10 databases (Targetscan, RNA22, PITA, PICTAR5, PICTAR4, RNAhybrid, miRWalk, miRDB, miRanda, and DIANAmT), and the miRTarBase, which comprises validated miRNA target interactions from experiments, were used to assess correlations between DEMs and DE-miRNAs [101].

### Analysis of data from the GTEX and TCGA databases

The R software (https://www.r-project.org/) with several publicly available packages was used for statistical analysis of data from the GTEX and TCGA databases. A human tissue–enriched protein expression map and a boxplot of genes were generated using the “gganatogram” and “ggpubr” models, respectively. For the genotypic correlation analysis, the *χ*² test or Fisher’s exact test (two-sided) was used.

### Western blot analysis

To evaluate protein expression, cells were harvested in RIPA buffer containing a protease inhibitor cocktail, and total protein was quantified using a bicinchoninic acid kit (Pierce, Rockford, IL, USA). Aliquots containing 8 μg total protein were separated by sodium dodecyl sulphate polyacrylamide gel electrophoresis and then electroblotted onto a 0.45-μm PV membrane (Immobilon™; Merck Millipore, Darmstadt, Germany). The membranes were blocked and then probed overnight with the primary antibodies anti-SCD1 (1:1000, #ab19862; Abcam, USA), anti-CDKN1A (1:10,000, #ab47300; Abcam, USA), anti-FOS (1:500, #ab184666; Abcam, USA), anti-EXO1 (1:2000, #ab95068; Abcam, USA), anti-PLS1 (1:2000, #ab236976; Abcam, USA), anti–β-catenin (1:5000, #ab32572; Abcam, USA), and anti–active β-catenin (1:500, #05-665; Merck Millipore). Results are expressed as means ± standard deviations of six independent experiments.

### Quantitative real-time PCR (qPCR)

Total RNA was isolated from cell cultures using TRIzol® reagent (Gibco/Life 270 Technologies, Thermo Fisher Scientific). RNA quantity and quality were detected by stem-loop quantitative RT-PCR (TaqMan probe method). Purified RNA was used for first-strand cDNA synthesis with M-MLV reverse transcriptase and primers according to the manufacturer’s instructions (Promega, Fitchberg, MA, USA). The primer sequences were designed by Primer Premier and the sequences were as follows: FOS forward 5’-GGAGATGTAGCAAAACGCAT-3’ and reverse 5’- GTTAATTCCAATAATGAACCCAA-3’; EXO1 forward 5’- GCGGCTGCAGTCGTATGG-3’ and reverse 5’-ATTTGCGCGGGTTCCTTG-3’; PLS1 forward 5’-TGGTTTGATTTTTTTGGTGTGT-3’ and reverse 5’- CAAGAGAGTGAACTTTGGGGT-3’; CDKN1A forward 5’- GAGCCTCCCTCCATCCCTA-3’ and reverse 5’- CCATCCCCTTCCTCACCTG-3’; miR-1908 forward 5’-CGGCGGGGACGGCGA-3’ and reverse 5’- CCGCAGGGTCCGAGGTATTC-3’; miR-203a forward 5’-CGCGCGGGAAAGAGGA-3’ and reverse 5’- AGTGCAGGGTCCGAGGTATT-3’; U6 (endogenous control) was amplified using forward 5’- CTCGCTTCGGCAGCACA-3’ and reverse 5’- AACGCTTCACGAATTTGCGT-3’.

### Transfection of miRNAs

The transfection of miRNAs was performed as previously described [19]. In brief, chemically synthesized miRNAs mimic and inhibitor (Gene Pharma (Shanghai, China) were used to augment and inhibit miR-203a and miR-1908 function. At 24h after seeding, cells were transfected with miR-203amimic, miR-1908mimic, miR-203ainhibitor, or miR-1908inhibitor for 24 h using the riboFECT™ CP Transfection Kit according to the manufacturer’s protocol (Ribobio, Guangzhou, China).

### Dual luciferase reporter assay

As mentioned above, the double luciferase reporter gene was determined [102]. In short, UM Chor1 cells were inoculated into a 96-well plate at the density of 1×104 cells per well 48 h after 3'-UTR plasmid co-transfection. The dual luciferase reporter assay system (Promega, Madison, WI, USA) was used to harvest cell lysates and firefly and detect renilla luciferase activities.

### Statistical analysis

Statistical analysis was performed using GraphPad Prism (version 7.0) software. Results are expressed as mean ± SD deviation of three or six independent experiments. Statistical significance was analyzed by two-tailed t-test or one-way analysis of variance. The difference was statistically significant at P < 0.05.

### Ethics approval and consent to participate

This study was approved by the Institutional Ethics Review Board of Shanghai General Hospital, Shanghai Jiao Tong University, Shanghai, China.

### Availability of data and materials

Please contact author for data requests.

## Supplementary Material

Supplementary Figures
