# Supplementary material for: MiR-1908/EXO1 and MiR-203a/FOS, regulated by scd1, are associated with fracture risk and bone health in postmenopausal diabetic women
Source: Aging (Albany NY). 2020 May 26;12(10):9549–84. doi: 10.18632/aging.103227 (PMC7288911; doi:10.18632/aging.103227)
Supplement: Supplementary Figures [file aging-12-103227-s001..pdf]

## SUPPLEMENTARY FIGURES

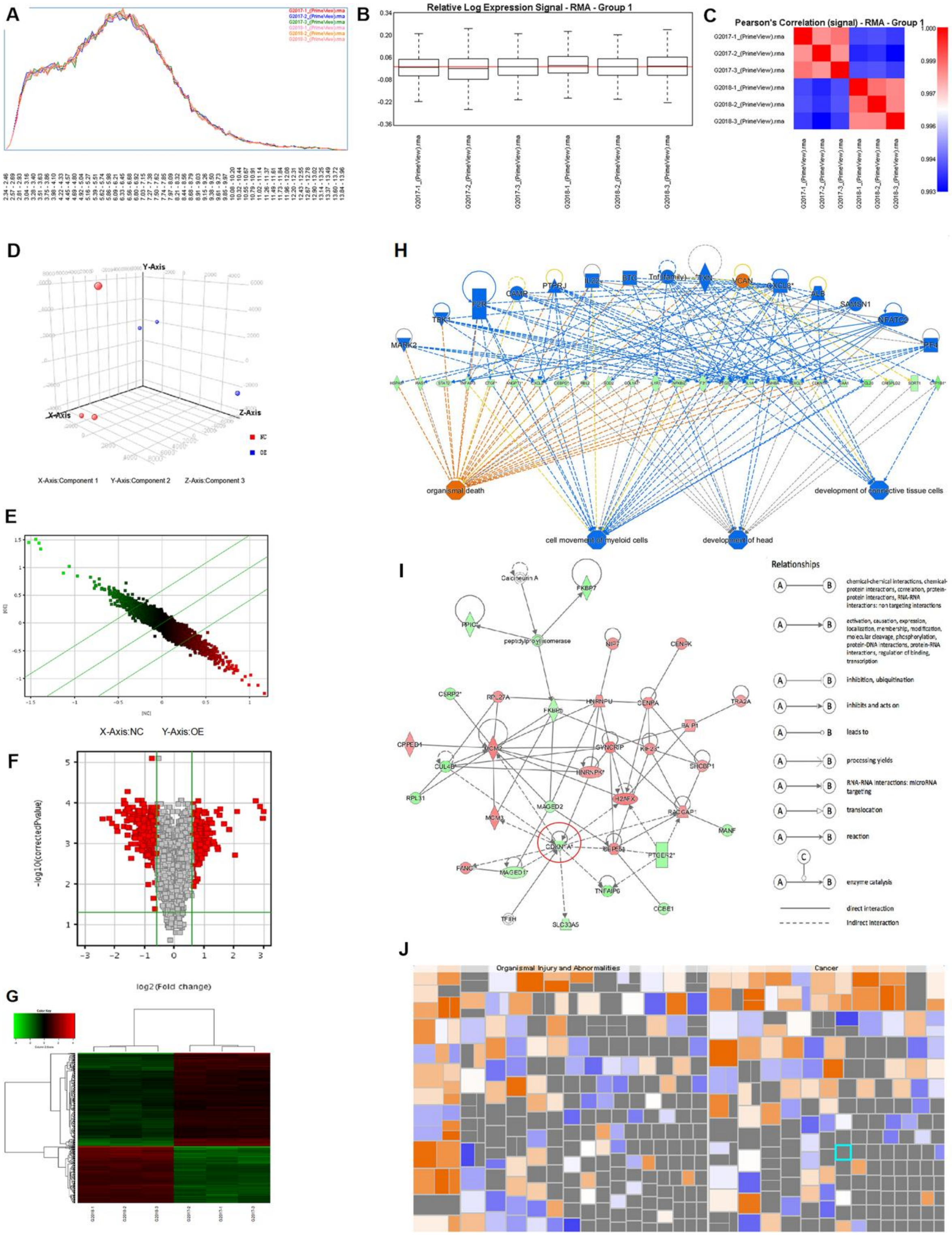


**Supplementary Figure 1. SCD1 overexpression induces expression changes in BM-MSCs.** (**A**) The signal value distribution graph shows the distribution of the expression values of all the chip probes. Each curve represents the number of probe statistic for a chip in different expression value intervals. The better the coincidence of the signal value distribution curve, the higher the reliability of the chip experiment. All the chips in this project are very reliable. (**B**) The relative logarithmic expression box plot shows the distribution of relative

logarithmic expression values for all chips. The closer the distribution of the relative logarithmic expression value box plot is, the higher the repeatability of the data. All samples in this project are highly reproducible. (**C**) The Pearson correlation coefficient (signal value) plot shows the level of signal value correlation between all chips and chips. There is a high correlation between G2017-1, G2017-2 and G2017-3 in this project and the correlation coefficient is greater than 0.95. (**D**) This three-dimensional Principal Component Analysis chart is used to indicate the similarity between groups and the degree of difference between groups. The higher the degree of aggregation between samples, the higher the similarity of samples. Obviously, there was a large difference between the overexpressed group and the control group in this project. (**E**) The scatter plot shows the distribution of signal values between the experimental and control groups on the Cartesian coordinate plane. Above the green line is a probe that overexpresses expression after overexpression of SCD1, and a probe that is downregulated after expression of SCD1 is expressed below the green line. (**F**) The volcano plot shows the differential gene distribution between the experimental and control groups. The abscissa represents the difference multiplier through the base 2 logarithmic transformation, the ordinate represents the corrected significance level through the base 10 logarithmic transformation; the red represents all probes with a difference multiple greater than 1.5 and a significance level less than 0.05. (**G**) The heat map of Differentially expressed micro-RNAs from the Differentially expressed mRNAs within the expression of SCD1. The clustering graph shows the aggregation of all samples and differential genes at the expression level. Red indicates that the signal value of the gene is relatively up regulated. Green indicates that the signal value of the gene is relatively downregulated, black indicates that the signal value of the gene is moderate, and gray indicates that the signal value of the gene is not detected. (**H**) The gene interaction network map shows a network of interactions from the defined functional area (Organ Development, Organ Morphology, Reproductive System Development and Function) between the Differentially expressed mRNAs within the expression of SCD - 1. (**I**) The regulatory effect network map shows the interaction between genes and regulators and functions in the dataset. (**J**) The disease and functional heat maps show the up-regulation and down-regulation of differential gene expression on the activation-inhibition of function and disease. Orange represents Z-score>0, blue represents Z-score<0, gray indicates no Z-score value; Z-score>2 indicates that the function is significantly activated, and Z-score<-2 indicates that the function is significantly inhibited. The related functions of this project have obvious activation: organismal death (2.715) promotion of cells, 2.639; the functions of significant inhibition are: survival of organism (-3.890), size of body, -3.162, etc.

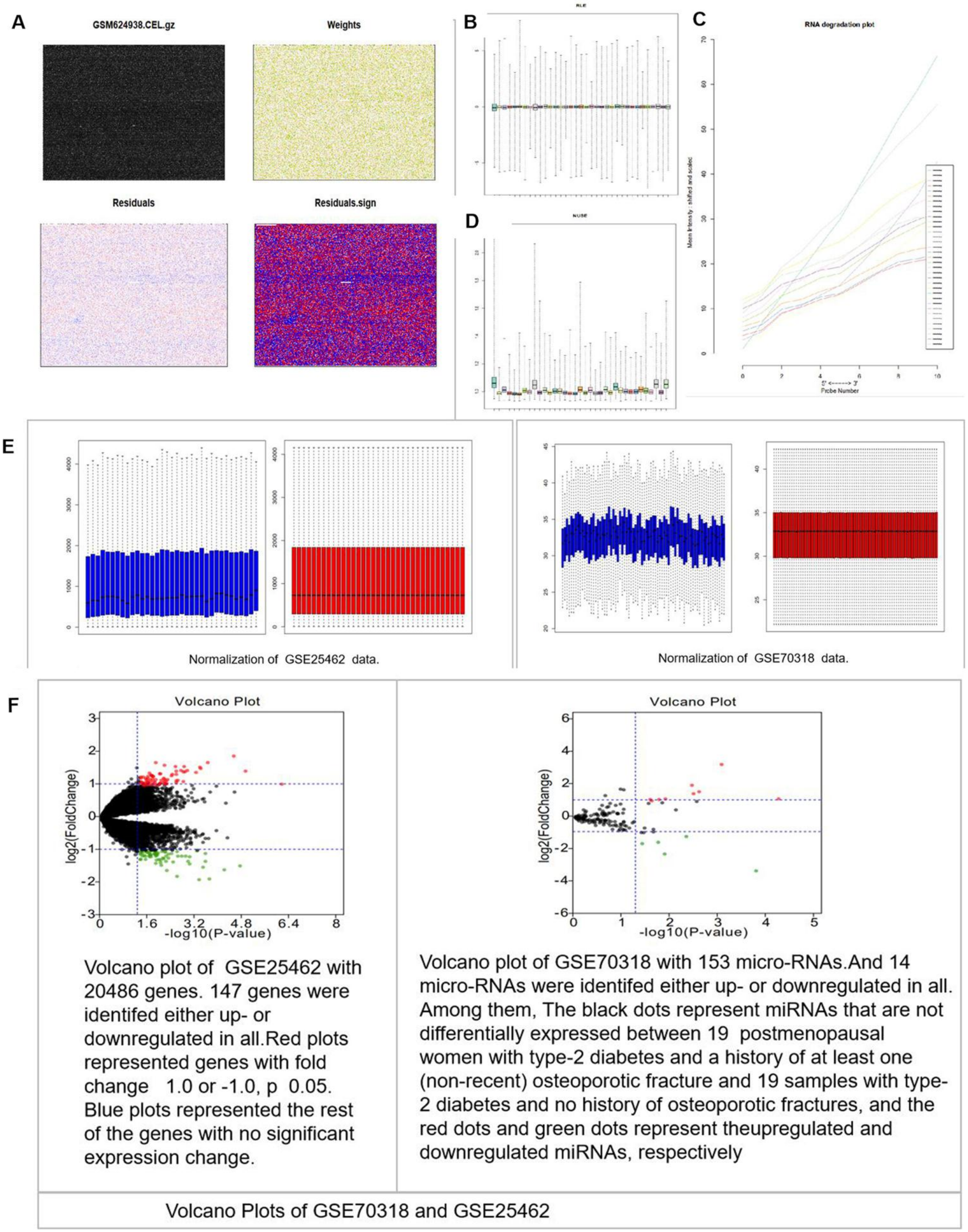


**Supplementary Figure 2. DE-miRNAs and DEMs selected from GEO datasets.** (**A**) Quality control of GSE25462 data by using the R language "affyPLM" package (grey-scale map, weighted graph, residuals plots, residual symbol diagram); (**B**) RLE graph: the logarithm of the

expressed value of a probe group in a sample which reflects the consistency of the parallel experiment divided by the median expressed by the probe group in all samples; (**C**) RNA degradation plot: RNA degradation began at the 5 'end, because the fluorescence intensity at the 5' end was much lower than that at the 3 'end; (**D**) NUSE graph: the logarithm of the standard deviation of the PM value of a probe group in a sample divided by the median of the PM value standard deviation of the probe group in each sample reflects that the consistency of parallel experiments is more sensitive than that of RLE; (**E**) Normalization of GSE25462 and GSE70318. Blue: data before normalization; Red: data after normalization; (**F**) Volcano Plots of GSE70318 and GSE25462.

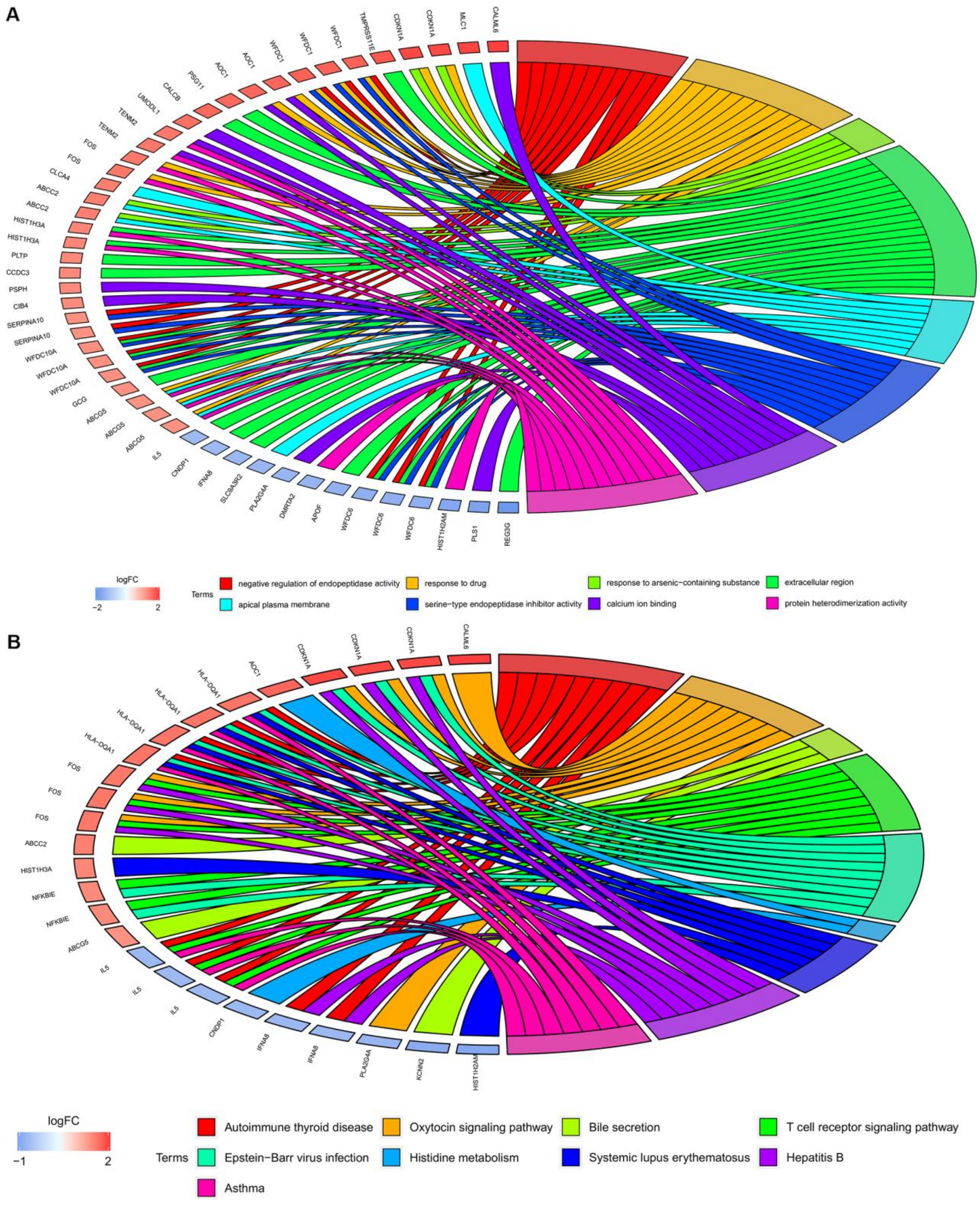


**Supplementary Figure 3. Circularly composited overviews of selected genes and their terms.** (**A**) GO functional enrichment of Differentially expressed mRNAs in GSE25462; (**B**) KEGG enrichment analysis of Differentially expressed mRNAs in GSE25462.

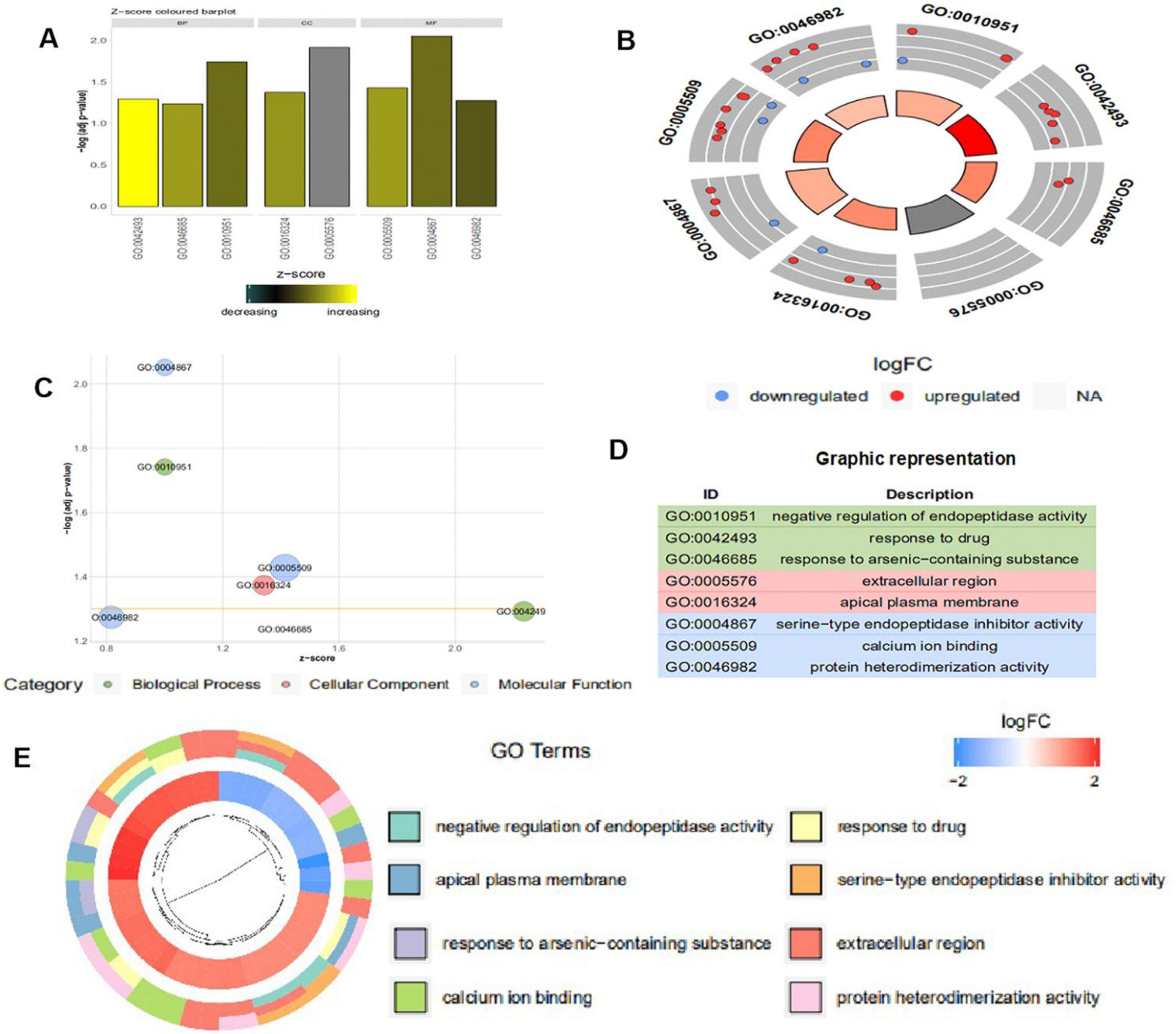


**Supplementary Figure 4. GO functional enrichment analysis of Differentially expressed mRNAs in GSE25462.** (**A**) Bar: Z-score coloured barplot of terms ordered alternatively by z-score or the negative logarithm of the adjusted p-value; (**B**) Circular plot: the plot combines gene expression and gene- annotation enrichment data. A subset of terms is displayed like the GOBar plot in combination with a scatterplot of the gene expression data. The whole plot is drawn on a specific coordinate system to achieve the circular layout; (**C**) Bubble plot: The x- axis of the plot represents the z-score. The negative logarithm of the adjusted p-value (corresponding to the significance of the term) is displayed on the y-axis. The area of the plotted circles is proportional to the number of genes assigned to the term; (**D**) Graphic representation of **A**, **B**, and **C**; (**E**) GOCluster: generates a circular dendrogram of the data clustering. The inner ring displays the color coded logFC while the outside one encodes the assigned terms to each gene.

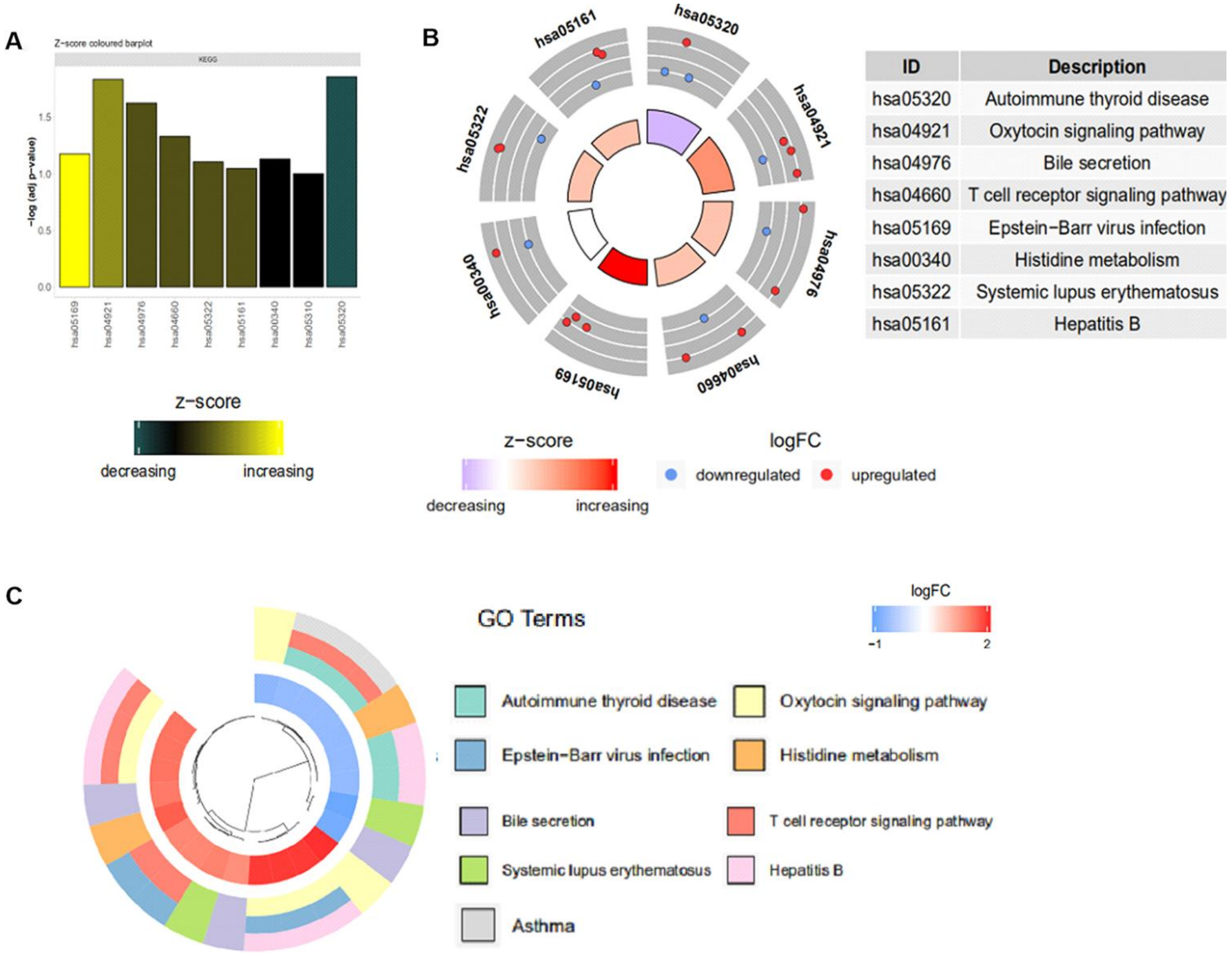


**Supplementary Figure 5. KEGG enrichment analysis of Differentially expressed mRNAs in GSE25462.** (**A**) Bar: Z-score coloured barplot of terms ordered alternatively by z-score or the negative logarithm of the adjusted p-value; (**B**) Circular plot: the plot combines gene expression and gene- annotation enrichment data. A subset of terms is displayed like the GOBar plot in combination with a scatterplot of the gene expression data. The whole plot is drawn on a specific coordinate system to achieve the circular layout; (**C**) GOCluster: generates a circular dendrogram of the data clustering. The inner ring displays the color coded logFC while the outside one encodes the assigned terms to each gene.

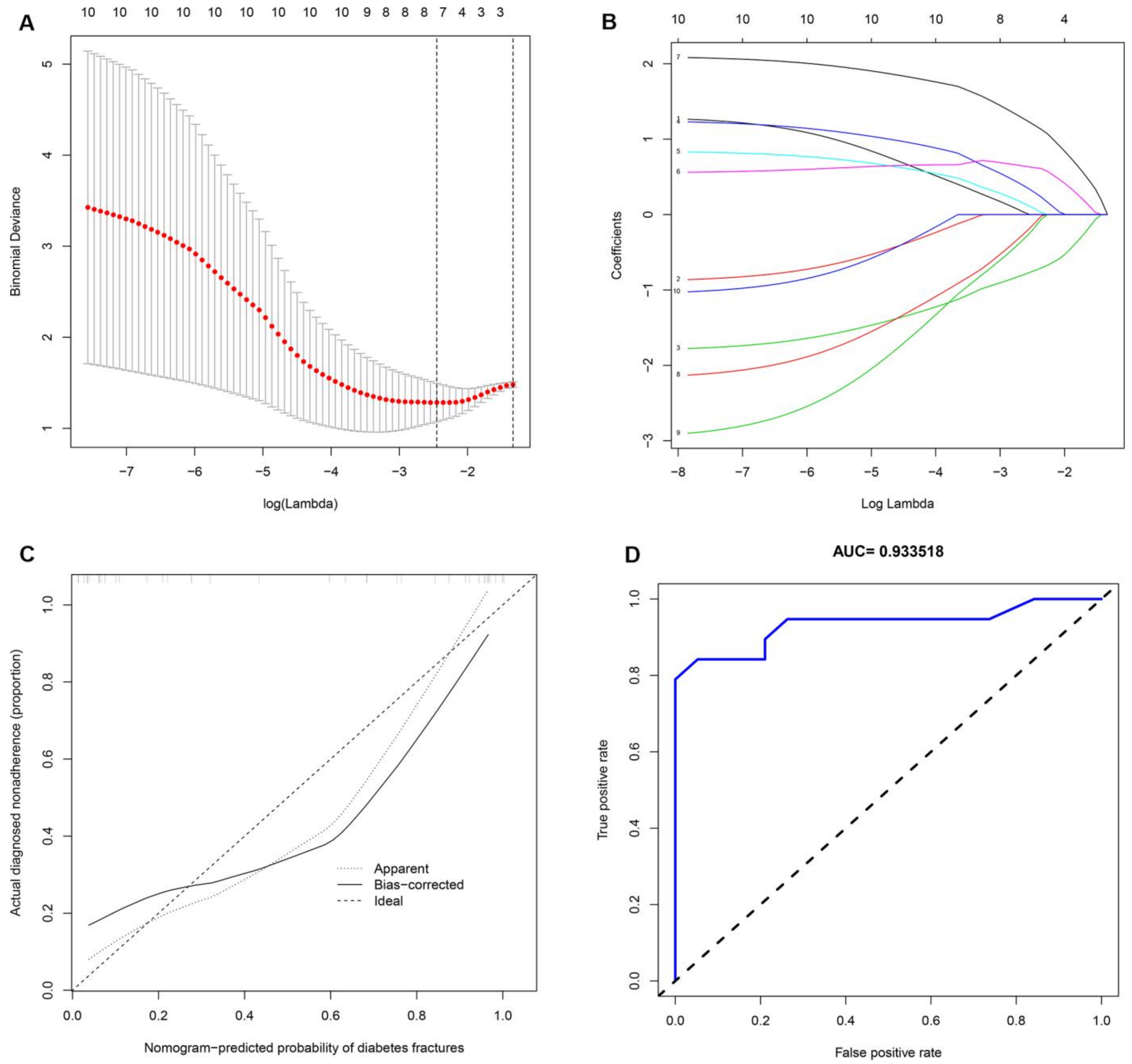


**Supplementary Figure 6. miRNAs selected and detected by predictive modeling of diabetic fracture risk.** (**A**) Differentially expressed micro-RNAs and demographic selection using the least absolute shrinkage and selection operator (LASSO) binary logistic regression model. Optimal parameter (lambda) selection in the LASSO model was performed via fivefold cross-validation using minimum criteria. The partial likelihood (binomial) deviance curve is plotted versus log(lambda). Dotted vertical lines indicate optimal values determined by the minimum criteria and 1 standard error of the minimum criteria. (**B**) LASSO coefficient profiles of the 10 features. The coefficient profile plot was produced against the log(lambda) sequence. The vertical line indicates the value selected using fivefold cross-validation, where the optimal lambda enabled the identification of seven features with nonzero coefficients. (**C**) Calibration curves for nomogram-based diabetic fracture prediction in the cohort. *X* axis, predicted fracture risk; *y* axis, actual diabetic fractures. The diagonal dotted line represents perfect prediction by an ideal model and the solid line represents the performance of the nomogram. (**D**) The area under the curve for the diabetic fracture nomogram (0.933518) indicates that a randomly chosen positive example will likely be ranked higher than a randomly chosen negative example.

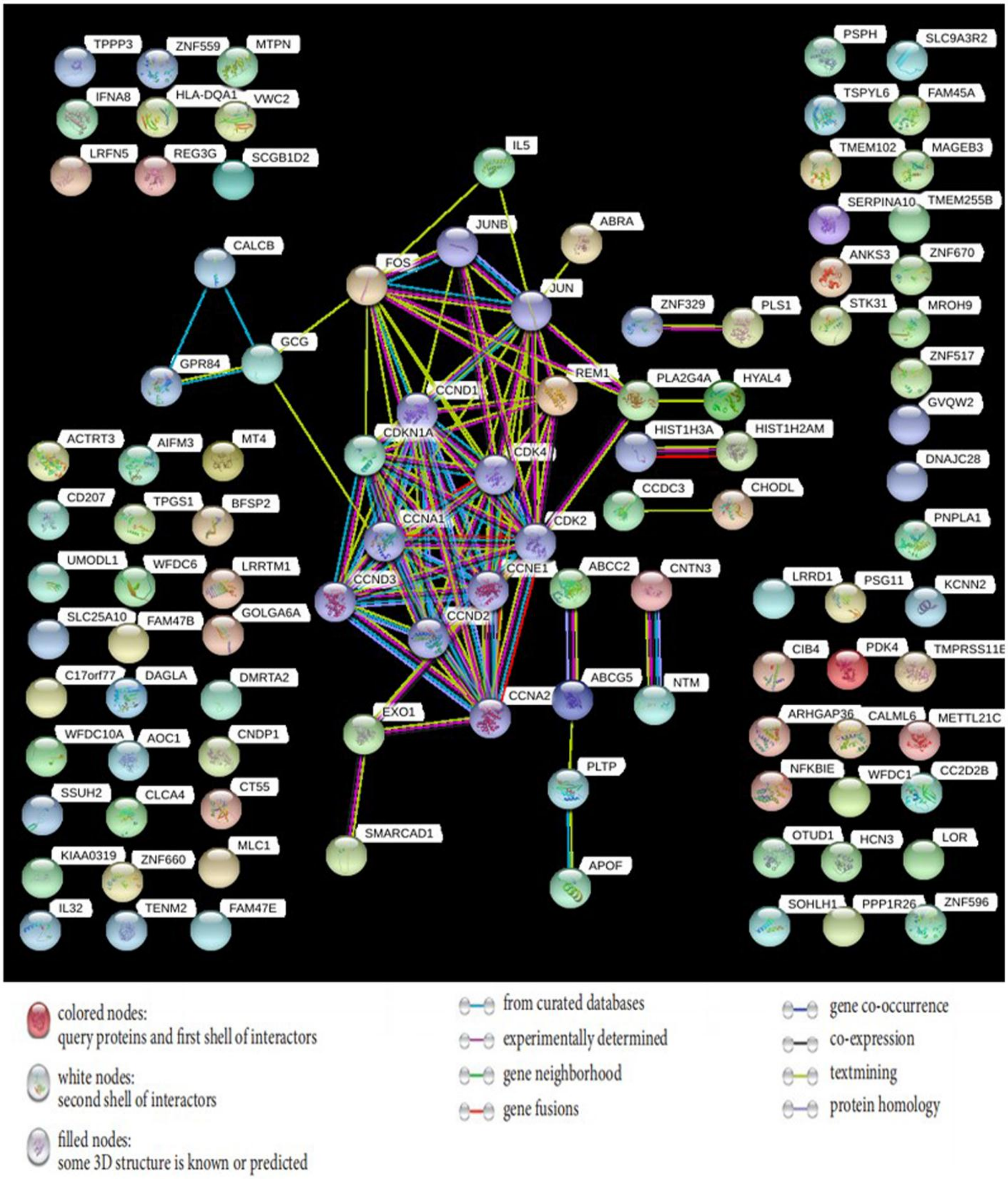


**Supplementary Figure 7. Construction of protein-protein interaction (PPI) network.** PPI enrichment, P-value=8.19e-0.
